# Therapeutic advances in non‐small cell lung cancer: Focus on clinical development of targeted therapy and immunotherapy

**DOI:** 10.1002/mco2.105

**Published:** 2021-12-14

**Authors:** Yuan Cheng, Tao Zhang, Qing Xu

**Affiliations:** ^1^ Laboratory of Aging Research and Cancer Drug Target State Key Laboratory of Biotherapy and Cancer Center National Clinical Research Center for Geriatrics West China Hospital Sichuan University Chengdu China; ^2^ Department of Oncology Shanghai Tenth People's Hospital Tongji University School of Medicine Shanghai China

**Keywords:** combination therapy, drug resistance, immunotherapy, non‐small cell lung cancer (NSCLC), targeted therapy

## Abstract

Lung cancer still contributes to nearly one‐quarter cancer‐related deaths in the past decades, despite the rapid development of targeted therapy and immunotherapy in non‐small cell lung cancer (NSCLC). The development and availability of comprehensive genomic profiling make the classification of NSCLC more precise and personalized. Most treatment decisions of advanced‐stage NSCLC have been made based on the genetic features and PD‐L1 expression of patients. For the past 2 years, more than 10 therapeutic strategies have been approved as first‐line treatment for certain subgroups of NSCLC. However, some major challenges remain, including drug resistance and low rate of overall survival. Therefore, we discuss and review the therapeutic strategies of NSCLC, and focus on the development of targeted therapy and immunotherapy in advanced‐stage NSCLC. Based on the latest guidelines, we provide an updated summary on the standard treatment for NSCLC. At last, we discussed several potential therapies for NSCLC. The development of new drugs and combination therapies both provide promising therapeutic effects on NSCLC.

## Graphical abstract

Based on the development of targeted therapy and immunotherapy, the treatment strategies for NSCLC have been profoundly changed. After evaluation of the incidental finding of nodule, patients are managed to routine follow‐up or diagnosis of NSCLC. Patients with NSCLC should receive molecular testing and PD‐L1 testing to make final treatment decision. For resectable NSCLC with targetable mutations, targeted therapy as adjuvant therapy shows promising effects. Immunotherapy has also been investigated as both neoadjuvant and adjuvant therapy. For unresectable advanced NSCLC with driver gene positive, targeted therapy and immunotherapy play an essential role in anticancer treatment. This review comprehensively presents the current landscape of targeted therapy and immunotherapy in NSCLC.

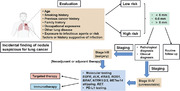

## 1 INTRODUCTION

Lung cancer is still the most common cancer worldwide and contributes to nearly one‐quarter cancer‐related deaths in 2021, more than 80% of which are directly caused by tobacco smoking. An additional 2.7% deaths are due to second‐hand smoke.^1^ As a major component of lung cancer, non‐small cell lung cancer (NSCLC) accounts for 80–85%, of which lung adenocarcinoma (LUAD) and lung squamous cell carcinoma (LUSC) are the most common subtypes.^2^ The incidence of lung cancer in developed countries has been declined during recent years, whereas the incidence and mortality rates in China have been significantly increased as a result of difference in lifestyle and development of economy.^3^ The mortality of lung cancer in China is estimated and may increase by approximately 40% from 2015 to 2030.^4^ Thus, the public health and therapeutic strategies for lung cancer, especially NSCLC, remain critical.

Tobacco smoking is directly associated with NSCLC and there are more than 50 carcinogens in the tobacco smoke.^5^ Effective tobacco control helps to decrease the incidence of lung cancer in the United State.^3^ Tobacco control is assumed to be a convenient and effective measure to lower the incidence and mortality of lung cancer. Other carcinogenic factors, such as bad lifestyle, genetic mutations, and family cancer history, also contribute to lung cancer. Except for cancer prevention, lung cancer screening is also crucial in detecting early‐stage patients, of which low‐dose computed tomographic (CT) screening reduces the mortality of lung cancer.^6^ Despite the high sensitivity of CT screening, high rates of false‐positive findings make the size thresholds important. According to the American National Comprehensive Cancer Network (NCCN) guidelines for NSCLC (2021), routine follow‐up by chest CT is required for low‐risk patients (i.e., patients without smoking history or other known risk factors) when the solid nodule(s) on CT ≥ 6 mm. Efficient CT screening for early detection is the second defender for fight against NSCLC.^7^

Surgery is recommended for early‐stage (stage I–II) NSCLC patients, whereas more than 70% of NSCLC are diagnosed as advanced stage (stage III–IV).^8^ Cytotoxic therapy, targeted therapy, and immunotherapy are essential for advanced‐stage NSCLC patients. During last decades, much progress has been made in the therapeutic strategies for advanced NSCLC, especially the development of targeted therapy and immunotherapy. NSCLC is a heterogeneous malignancy with large‐scale genomic studies profiling a diversity of driver gene mutations. Genetic features are the basis of “precision and personalized medicine.” For now, several small molecular tyrosine kinase inhibitors (TKIs), which target *EGFR (epidermal growth factor receptor)* mutation, rearrangements in *ALK (anaplastic lymphoma kinase)*, fusions in *ROS1(ROS proto‐oncogene 1)*, *BRAF (v‐Raf murine sarcoma viral oncogene homolog B)* V600E, *NTRK (neurotrophic tyrosine receptor kinase)1/2/3* gene fusion, *MET (mesenchymal‐epithelial transition)* exon 14 skipping, and *RET (rearranged during transfection)* rearrangement, have been approved by the U.S. Food and Drug Administration (FDA) for the treatment of driven gene mutation‐positive NSCLC patients. Because of comprehensive genomic profiling, other genetic aberrances in NSCLC, such as mutations in Kirsten rat sarcoma (*KRAS*), amplification of human epidermal growth factor receptor‐2 *(HER2)*, and other genotypes of the driver genes, have been thought highly targetable and investigated in preclinical and clinical trials.^9^ Of note, compared with LUAD, LUSC rarely has *EGFR* mutation, *ALK* rearrangements, or *ROS1* fusions, but usually gets alterations in *RTKs, CDKN2A, PTEN, MLL2, HLA‐A, NOTCH1*, and *RB1*.^9^ Immunotherapy is another treatment strategy that has significantly prolonged the survival of NSCLC patients, especially those driver gene mutation‐negative NSCLC patients. Monoclonal antibodies (mAbs) targeting programmed cell death‐1 (PD‐1), programmed cell death ligand‐1 (PD‐L1), and cytotoxic T‐lymphocyte‐associated antigen‐4 (CTLA‐4) immune checkpoints have been approved for the treatment of a variety of cancers, including NSCLC. Five immune checkpoint inhibitors (ICIs) have been approved by FDA, including nivolumab and pembrolizumab (anti‐PD‐1 antibodies), atezolizumab and durvalumab (anti‐PD‐L1 antibodies), and ipilimumab (anti‐CTLA‐4 antibody).

Despite the development of the therapeutic strategies and improved survival for NSCLC, some major concerns remain challenging, such as the resistance to targeted therapy and immunotherapy, optimal combinations of the current treatment regimens, and investigation for new potential targets. Besides, with rapid development of targeted therapy during recent years, several breakthrough TKIs have been approved clinically. Based on the clinical management of NSCLC, we provide an overview for the treatment of advanced NSCLC and focus on targeted therapy and immunotherapy. Meanwhile, the important completed and ongoing clinical trials of both targeted therapy and immunotherapy are summarized in this review.

## 2 CLINICAL MANAGEMENT OF NSCLC

**FIGURE 1:**
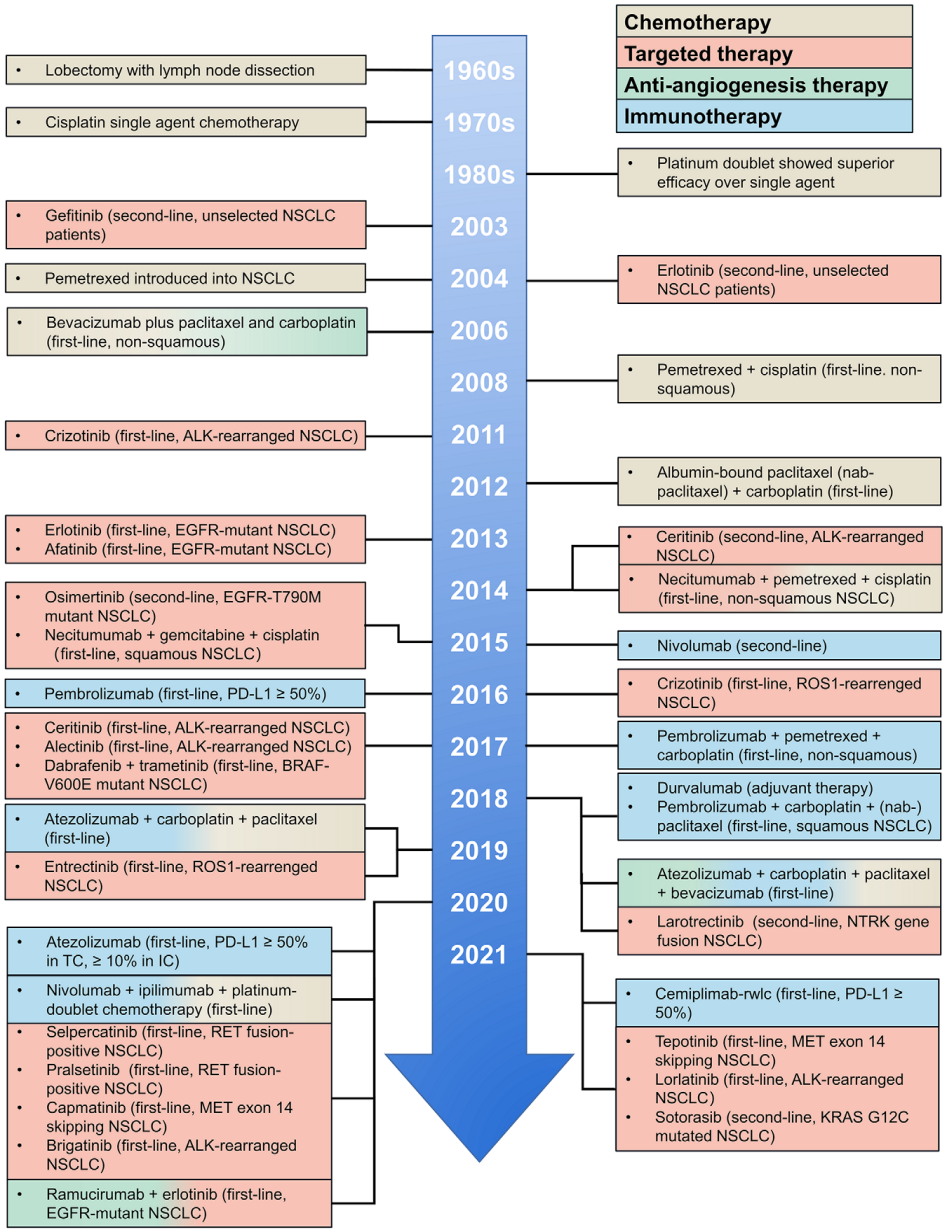
Timeline illustrating the development of treatment strategies for NSCLC. Surgery and cytotoxic chemotherapy have been introduced to NSCLC in 1960s and 1970s, for the first‐time improving prognosis of NSCLC. Antiangiogenesis therapy for nonsquamous NSCLC was approved by FDA in 2006, limited by its insufficient efficacy as monotherapy. The first‐line use of ALK TKIs and EGFR TKIs renewed the treatment strategies for NSCLC in early 2010s. Immunotherapy has developed rapidly during last 5 years. Pembrolizumab in combination with chemotherapy, approved in 2017, brings new hope for patients without targetable mutations. The combination therapies are presented in mixed colors

With the introduction of surgery and cytotoxic chemotherapy, the prognosis of NSCLC patients was improved for the first time. Through the development of molecular biology, new therapies, such as antiangiogenesis therapy, targeted therapy, and immunotherapy, have yielded encouraging therapeutic effects in advanced NSCLC. Remarkable changes have been made in the treatment of NSCLC during last decades (Figure 1).

### 2.1 Molecular testing for analysis

NSCLC is a molecularly heterogeneous disease, which makes early identification of tumor genotype critical. Companion diagnostic assays are usually released by the U.S. FDA for approval of targeted agents, in order to identify essential genomic alterations before initiating therapy.^10^, ^11^, ^12^ *EGFR, ALK*, and *ROS1* are initially recommended as three targetable oncogenic drivers, which must be tested if the tissue is limited for next‐generation sequencing (NGS) panel.^13^ NCCN recommends that all patients should be screened for *EGFR*, *ALK*, *KRAS*, *ROS1*, *BRAF*, *NTRK1/2/3*, *MET*, *RET*, and PD‐L1 expression. DNA sequencing is a traditional way to identify genomic mutations, which requires relatively enriched tumor cells. Mutation‐specific polymerase chain reaction (PCR) kits have been used in clinical laboratory for its high sensitivity with only 1–5% tumor cells.^11^ However, this method is limited by its narrow spectrum of mutations.^14^, ^15^ Oncogenic fusion mutation can be detected by fluorescence in situ hybridization (FISH) or PCR; however, these two methods are limited by the capacity to identify fusion partners.^16^, ^17^ Identification of fusion partners is essential in the treatment decision making.^18^, ^19^, ^20^, ^21^ For now, NGS has become a typical molecular testing method and is able to analyze the information from DNA and RNA.^16^, ^17^ RNA NGS is also capable to identify the fusion partners of *ALK*, *ROS1*, *RET*, and *NTRK*.^22^, ^23^, ^24^, ^25^ Liquid biopsies, another type of detecting methods approved by the U.S. FDA, are used to detect circulating tumor DNA (ctDNA).^26^ Though tissue biopsy remains the gold standard for diagnosis, liquid biopsy offers a way of continent and early diagnostic by body fluids.^27^ When tumor tissue is limited, a ctDNA assay can be used to identify certain genomic mutations, including *EGFR*.^28^ Immunohistochemistry (IHC) is a substitute for molecular testing, especially for ALK, ROS1, and NTRK.^29^, ^30^ For example, Ventana *ALK* D5F3 CDx Assay is the only IHC test approved by the U.S. FDA for ALK inhibitors.^31^, ^32^ Molecular testing becomes more and more important in clinical practice. Choosing the appropriate methods for a rapid diagnosis is crucial in making treatment decision for NSCLC patients.

### 2.2 Treatment for early‐stage NSCLC

**FIGURE 2:**
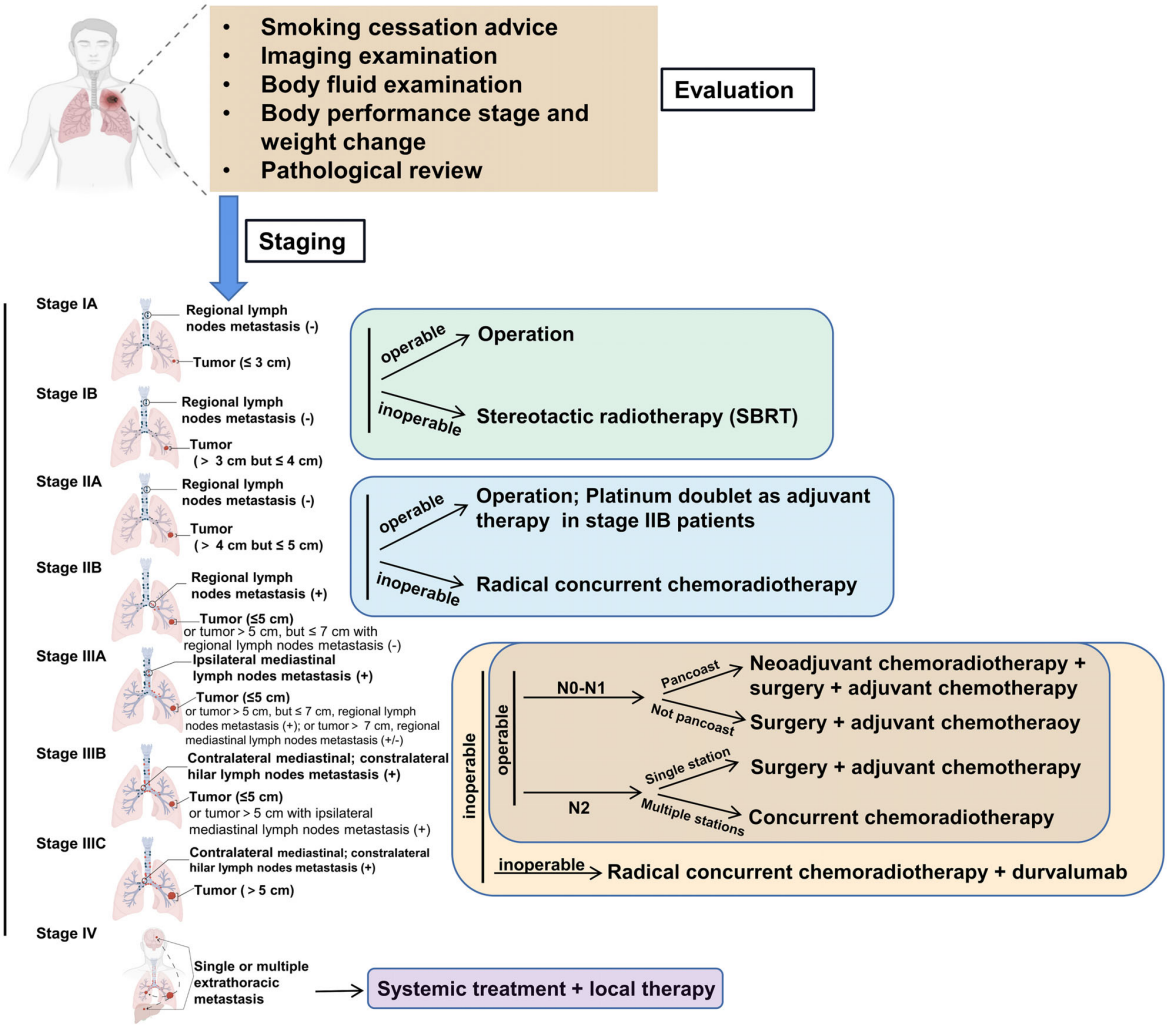
Treatment algorithm for NSCLC patients at early stage. Surgery is recommended for early‐stage NSCLC patients. For patients at stage IIA–IIIB, adjuvant therapy is required. Locally advanced or metastatic NSCLC should receive systemic therapy. The staging strategy is based on the guideline of National Comprehensive Cancer Network (NCCN) (2021)^49^

The treatment strategy for early‐stage NSCLC is based on surgery (Figure 2). Surgery is strongly recommended for patients at stage I–II, which offers an optimal chance to fight against the disease.^7^ Radical radiotherapy is another potentially curative treatment for localized NSCLC.^33^ For unresectable stage II NSCLC patients, concurrent chemoradiotherapy is recommended.^34^ Perioperative chemotherapy also contributed to a better survival for early‐stage patients.^35^ The efficacy of postoperative cisplatin‐based chemotherapy was confirmed by a pooled analysis, especially those at stage II and III.^36^ Adjuvant therapy of radiation is still under debt. In the LungArt study (IFCT‐0503, UK NCRI, and SAKK), postoperative radiation (PORT) demonstrated 3‐year disease‐free survival (DFS) of 47.1% in the PORT arm and 43.8% in the control arm among patients with resected N2‐positive NSCLC (stage III, lymph node‐positive) tumors, indicating no significant difference.^37^ Meanwhile, the role of targeted therapy or immunotherapy in the treatment for early‐stage patients has not been well defined.^38^, ^39^ For patients with stage IIB–IIIA or high‐risk stage IB–IIA, if the diver gene mutation is ensured by molecular testing, targeted therapy could be applied as adjuvant treatment.^40^, ^41^ Additionally, patients with specific gene mutation show worse prognosis than patients with wild‐type genotype.^42^ *EGFR* mutation‐positive NSCLC, targeted therapy as adjuvant treatment, has improved the survival of NSCLC patients. For instance, the 24‐month DFS is 89% in osimertinib group versus 52% in the placebo group, with reduced local relapse and metastasis.^43^ Recently, immunotherapy also showed impressive therapeutic effects. Atezolizumab as adjuvant therapy significantly improved the DFS of stage II–IIIA NSCLC.^44^ In 2021, atezolizumab was approved by FDA as an additional, or adjuvant, treatment for NSCLC patients (stage II–IIIA) received surgery or chemotherapy.^45^ Durvalumab also showed increased 5‐year survival in unresectable stage III NSCLC patients who have not progressed after chemotherapy.^46^ ALCHEMIST (Adjuvant Lung Cancer Enrichment Marker Identification and Sequencing Trial), an ongoing large‐scale trial conducted by the National Cancer Institute, contains four important components: biomarker analysis for high‐risk resectable NSCLC (A151216), adjuvant nivolumab for NSCLC without *EGFR* or *ALK* mutation (EA5142), and adjuvant EGFR (A081105) or ALK (E4512) TKIs for NSCLC with *EGFR* or *ALK* mutation.^47^, ^48^ This clinical trial will provide valuable answers on ways to selecting high‐risk early‐stage NSCLC and on the efficacy of targeted therapy or immunotherapy as adjuvant therapy.

### 2.3 Treatment for locally advanced or metastatic NSCLC (advanced NSCLC)

**FIGURE 3:**
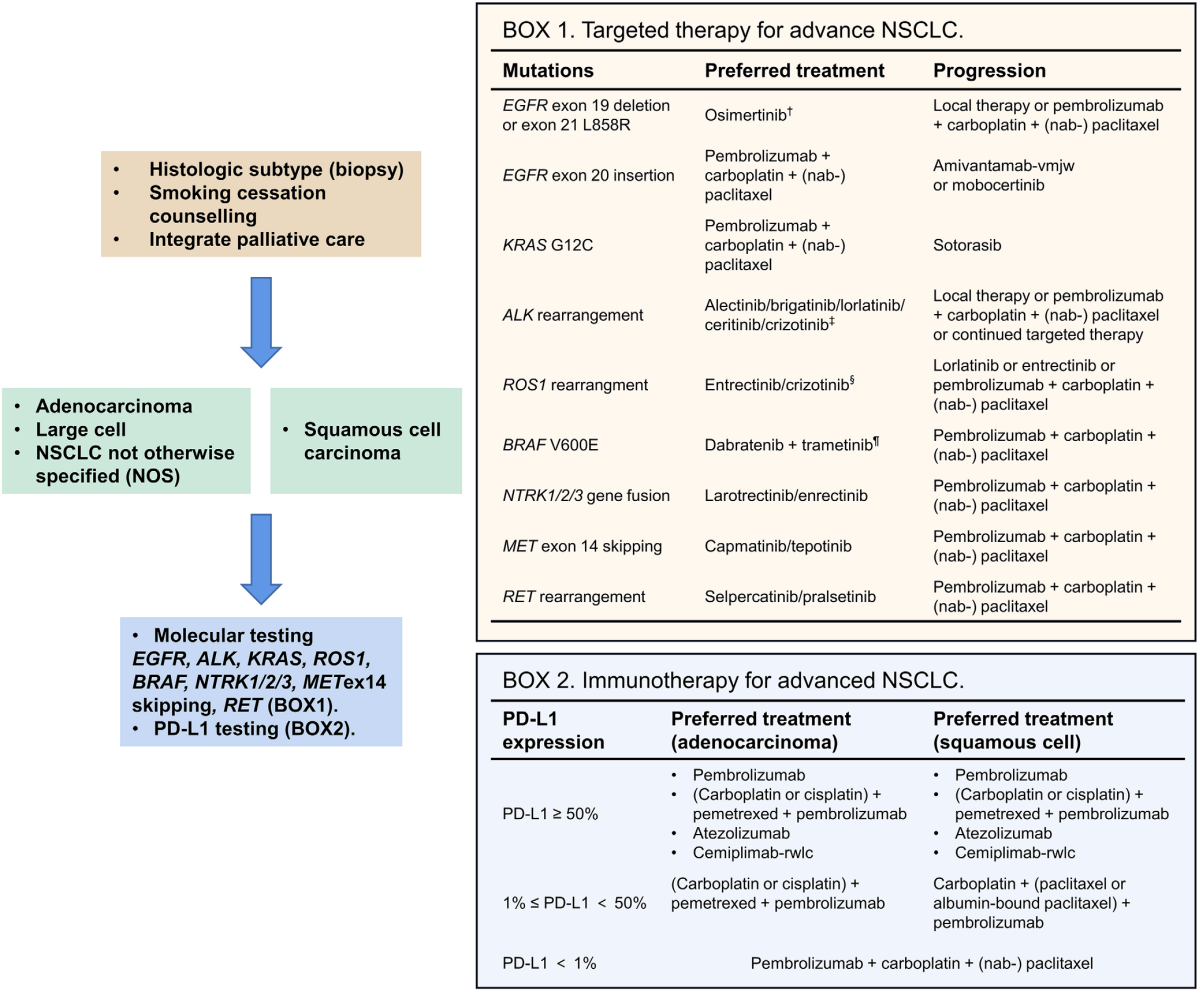
Treatment algorithm for advanced NSCLC. Advanced NSCLC consists of metastatic NSCLC and unresectable locally advanced NSCLC. *EGFR, ALK, KRAS, ROS1, BRAF, NTRK1/2/3, MET, RET*, and PD‐L1 expressions are included in the molecular testing. For patients without targetable mutations, pembrolizumab + carboplatin + (nab‐) paclitaxel is recommended. ^†^For patients with *EGFR* exon 19 deletion or exon 21 L858R, erlotinib, afatinib, gefitinib, and dacomitinib are also recommended. However, when the disease progresses on these TKIs, patients are recommended for a second molecular testing for T790M mutation test. ^‡^Patients who are intolerant to crizotinib may be switched to ceritinib, alectinib, or brigatinib. ^§^Entrectinib is recommended for patients with CNS metastasis. ^¶^Single‐agent vemurafenib is a treatment option if the combination of dabrafenib + trametinib is not tolerated

NSCLC patients in advanced phases usually lose the chance for surgery. For advanced NSCLC, combination chemotherapy of 4–6 cycles followed by observation is used to be treated as standard care.^50^, ^51^ The development of molecular biologic methods contributes to the identification of various subgroups of NSCLC. Basically, a newly diagnostic advanced NSCLC patients should receive molecular testing, PD‐L1 testing, and performance status scoring. Patients with good performance status might receive more aggressive treatment and benefit more. Around 70% of advanced NSCLC patients have a chance for targeted therapy or immunotherapy.^52^ For patients without targetable mutations or negative expression of PD‐L1, the standard first‐line treatments differ from the histological types. Patients with adenocarcinoma, large cell, or NSCLC not otherwise specified should receive pembrolizumab plus carboplatin (or cisplatin) and pemetrexed, whereas patients with squamous cell carcinoma receive pembrolizumab plus carboplatin and (nab‐) paclitaxel (Figure 3). It is assumed that up to 16% of patients with squamous and 15% with nonsquamous advanced NSCLC surviving for 5 years or more, due to combination therapy with immunotherapy.^53^ A comprehensive understanding of the current targeted therapy and immunotherapy is helpful in developing clinical treatments.

## 3 TARGETED THERAPY FOR NSCLC

Patients with advanced NSCLC benefit a lot from the development of targeted therapy. It is estimated that more than 65% of patients with advanced NSCLC have a potentially targetable genomic alteration.^52^ Based on the knowledge of genomic alterations, targeted therapy becomes the first‐line treatment for selected NSCLC patients, including inhibitors for EGFR, ALK, ROS1, BRAF, NTRK1/2/3, MET, and RET (Table 1). Of note, LUSC rarely have those genomic alterations.

**TABLE 1:** Targeted therapy in driver gene‐positive advanced NSCLC patients

Target	Inhibitor	Indications	Status	Company	Key clinical trials
EGFR (first generation)					
	^†^Geftinib (Iressa)	Advanced NSCLC with *EGFR* exon 19 deletion or exon 21 L858R mutations	FDA, Jul 2015 (first line)	AstraZeneca	IPASS, NCT00322452^54^
	Erlotinib (Tarceva)	Advanced NSCLC with *EGFR* exon 19 deletion or exon 21 L858R mutations	FDA, Nov 2013 (first line)	Genentech	OPTIMAL, NCT00874419^55^
	Icotinib (Conmana)	Advanced NSCLC with *EGFR* exon 19 deletion or exon 21 L858R mutations	NMPA, Nov 2014 (first line)	Beta Pharma	ICOGEN, NCT01040780^56^
EGFR (second generation)					
	^†^Afatinib (Gilotrif)	Advanced NSCLC with *EGFR* exon 19 deletion or exon 21 L858R mutations	FDA, Jul 2013 (first line)	Boehringer Ingelheim	LUX‐Lung 2, NCT00525148^57^; LUX‐Lung 3, NCT00949650^58^; LUX‐Lung 6, NCT01121393^59^
	^†^Dacomitinib (Vizimpro)	Advanced NSCLC with *EGFR* exon 19 deletion or exon 21 L858R mutations	FDA, Nov 2015 (first line)	Pfizer	ARCHER 1050, NCT01774721^60^
EGFR (third generation)					
	^†^Osimertinib (Tagrisso)	Advanced NSCLC with *EGFR* T790M mutation Advanced NSCLC with *EGFR* exon 19 deletion or exon 21 L858R mutations	FDA, Nov 2015 (first line) FDA, Dec 2020 (first line)	AstraZeneca	FLAURA, NCT02296125^61^; ADAURA, NCT02511106^62^
	Aulmonertinib (Ameile)	Advance NSCLC patients with *EGFR* T790M, who got disease progression on or after EGFR TKI therapy	NMPA, Mar 2020 (second line)	EQRx and Hansoh Pharma	APOLLO, NCT02981108^63^
	Furmonertinib (Alflutinib)	Advance NSCLC patients with *EGFR* T790M, who got disease progression on or after EGFR TKI therapy	NMPA, Mar 2020 (second line)	Shanghai Allist Pharmaceuticals	ALSC003, NCT03452592^64^
Anti‐EGFR antibody					
	Necitumumab (Portrazza)	In combination with pemetrexed and cisplatin, for patients with metastatic nonsquamous NSCLC In combination with gemcitabine and cisplatin, for patients with metastatic squamous NSCLC	FDA, Nov 2014 (first line) FDA, Nov 2015 (first line)	Eli Lilly	INSPIRE, NCT00982111^65^ SQUIRE, NCT00981058^66^
EGFR (exon 20 insertion)					
	Amivantamab‐vmjw (Rybrevant)	Advanced exon 20 insertion‐positive NSCLC patients progressed after platinum‐based chemotherapy	FDA, May 2021 (second line)	Janssen Biotech	CHRYSALIS, NCT02609776^67^
	Mobocertinib (Exkivity)	Advanced exon 20 insertion‐positive NSCLC patients progressed after platinum‐based chemotherapy	FDA, Sep 2021 (second line)	Takeda Pharmaceuticals	Study 101, NCT02716116^68^
ALK					
	^†^Crizotinib (Xalkori)	Advanced NSCLC with *ALK* rearrangement	FDA, Aug 2011 (first line)	Pfizer	PROFILE 1001, NCT00585195^69^; PROFILE 1005, NCT00932451^70^
	^†^Ceritinib (Zykadia)	Advanced NSCLC patients with *ALK* arrangements	FDA, May 2017 (first line)	Novartis	ASCEND‐4, NCT01828099^71^
	^†^Alectinib (Alecensa)	Advanced NSCLC patients with *ALK* arrangements	FDA, Nov 2017 (first line)	Hoffmann‐La Roche and Genentech	ALUR, NCT02604342^72^; ALEX, NCT02075840^73^
	^†^Brigatinib (Alunbrig)	Advanced NSCLC patients with *ALK* arrangements	FDA, May 2020 (first line)	ARIAD	ALTA 1L, NCT02737501^74^
	^†^Lorlatinib (Lorbrena)	Advanced NSCLC patients with *ALK* arrangements	FDA, Mar 2021 (first line)	Pfizer	Study B7461001, NCT01970865^75^; Study B7461006, NCT03052608^76^
ROS1					
	^†^Crizotinib (Xalkori)	Advance *ROS1*‐positive NSCLC	FDA, Mar 2016 (first line)	Pfizer	PROFILE 1001, NCT00585195^69^
	^†^Entrectinib (Rozlytrek)	Advance *ROS1*‐positive NSCLC	FDA, Aug 2019 (first line)	Genentech	STARTRK‐1, NCT02097810; STARTRK‐2, NCT02568267; ALKA‐372‐001, EudraCT number, 2012‐000148‐88^77^
BRAF					
	^†^Dabrafenib (Tafinlar)	In combination with trametinib for advance *BRAF* V600E‐positive NSCLC	FDA, Jun 2017 (first line)	Novartis	BRF113928, NCT01336634^78^
KRAS					
	Sotorasib (Lumakras)	*KRAS* G12C‐mutated advanced NSCLC who have received at least one prior systemic therapy	FDA, May 2021 (second line)	Amgen	CodeBreaK 100, NCT03600883^79^
RET					
	^†^Selpercatinib (Retevmo)	Advanced *RET* fusion‐positive NSCLC	FDA, May 2020 (first line)	Eli Lilly	LIBRETTO‐001, NCT03157128^80^
	^†^Pralsetinib (Gavretotm)	Advanced *RET* fusion‐positive NSCLC	FDA, Sep 2020 (first line)	Blueprint Medicines Corporation	ARROW, NCT03037385^81^
MET					
	^†^Capmatinib (Tabrecta)	Advanced NSCLC patients with *MET* exon 14 skipping alterations	FDA, May 2020 (first line)	Novartis	GEOMETRY mono‐1 trial, NCT02414139^82^
	^†^Tepotinib (Tepmetko)	Advanced NSCLC patients with *MET* exon 14 skipping alterations	FDA, Feb 2021 (first line)	Merck KGaA	V ISION, NCT02864992^83^
	Crizotinib (Xalkori)	Advanced *MET* exon 14 skipping‐positive NSCLC patients progressed after platinum‐based chemotherapy	FDA, May 2018 (second line)	Pfizer	PROFILE 1001 study, NCT00585195^69^
	Savolitinib (Orpathys)	Advanced NSCLC patients with *MET* exon 14 skipping alterations who have progressed after or who are unable to tolerate platinum‐based chemotherapy	NMPA, Jun 2021 (second line)	HUTCHMED and AstraZeneca	NCT02897479^84^
NTRK 1/2/3					
	^†^Entrectinib (Rozlytrek)	Solid tumors that have a *neurotrophic receptor tyrosine kinase (NTRK)* gene fusion without a known acquired resistance mutation	FDA, Aug 2019 (second line)	Genentech Inc	STARTRK‐1, NCT02097810; STARTRK‐2, NCT02568267; ALKA‐372‐001, EudraCT number, 2012‐000148‐88^85^
	^†^Larotrectinib (VITRAKVI)	Solid tumors that have a *neurotrophic receptor tyrosine kinase (NTRK)* gene fusion without a known acquired resistance mutation	FDA, Nov 2018 (second line)	Loxo Oncology Inc. and Bayer	LOXO‐TRK‐14001, NCT02122913^86^; SCOUT, NCT02637687^87^; NAVIGATE, NCT02576431^88^
	Repotrectinib (TPX‐0005)	Solid tumors that have a *neurotrophic receptor tyrosine kinase (NTRK)* gene fusion without a known acquired resistance mutation	FDA, Oct 2021 (second line)	Turning Point Therapeutics, Inc	TRIDENT‐1, NCT03093116^89^

*Note*: Inhibitors with † are recommended as first‐line treatment for advanced NSCLC with corresponding mutations, according to the NCCN guideline 2021.

Abbreviations: FDA, the U.S. Food and Drug administration; NMPA, Chinese National Medical Products Administration.

### 3.1 EGFR

There are 58 receptor tyrosine kinases (RTKs) in humans, and the epidermal growth factor receptor (EGFR, HER1, and ErbB1) is one of the first RTKs regarded as an anticancer target and brings significant improvement in the survival of NSCLC patients.^90^ EGFR belongs to tyrosine kinase type I receptors family that also includes human epidermal growth factor receptor 2 (HER2 and ErbB2), HER3 (ErbB3), and HER4 (ErbB4). EGFR is normally expressed on the surface of epithelial cells and regulates cell growth, survival, invasion, and angiogenesis.^91^ The *EGFR* gene is located on the short arm of chromosome 7 (7p11.2) and contains 28 exons and 27 introns. Mutations clustering around the ATP‐binding pocket of the tyrosine kinase domain lead to constituent, ligand‐independent activation of EGFR, which results in inappropriate activation of the antiapoptotic Ras signaling pathway and abnormal proliferation of cancer cells.^92^, ^93^ *EGFR* mutations in NSCLC occur in approximately 40% of Asian patients, about 10% of non‐Asian patients, and most of them are LUAD, young females, and nonsmoker.^94^, ^95^ The most common activating mutations of *EGFR* in NSCLC (approximately 85%) include exon 19 deletions and a point mutation on exon 21 (Leu858Arg, L858A), known as sensitizing mutations, which are responsible for oral TKIs targeting EGFR. Other mutations like exon 20 insertions and point mutations on exon 18 are less common.^92^ Compared with other mutations, patients with exon 19 deletion usually benefit more from targeted therapy and show a better survival.^96^, ^97^ Mutations at *EGFR* exon 20 are a heterogeneous group, some of which are predictions for the response to EGFR TKIs, such as T790M mutation.^98^, ^99^ Therefore, detailed knowledge of the specific alteration is required during genomic testing of *EGFR*. Results from recent data showed that patients without sensitizing *EGFR* mutation should not receive EGFR TKIs in any line of therapy.^100^

Three generations of EGFR TKIs have been used in clinical applications. First‐generation EGFR TKIs, including gefitinib, erlotinib, and icotinib, reversibly bind to EGFR and competitively inhibit the binding of ATP to tyrosine kinase domain. Gefitinib and erlotinib were first approved by the U.S. FDA for treatment of patients with advanced NSCLC (unselected), who have failed in standard chemotherapy.^101^ However, the use of these two TKIs in unselected NSCLC patients remained controversial.^93^, ^102^ In 2009, researchers found that nonsmokers or former light smokers in East Asia benefited more from gefitinib, especially those with *EGFR* mutation.^54^ Subsequently, several large clinical trials, including IPASS, WJTOG3405, NEJGSG002, OPTIMAL, EURTAC, and ENSURE, confirmed that gefitinib and erlotinib were superior to chemotherapy in NSCLC patients with *EGFR* mutations (especially sensitizing mutation, exon 19 deletion and exon 21 L858R) in terms of progression‐free survival (PFS), objective response rate (ORR), and quality of life, whereas these EGFR TKIs showed no advantage in patients without such mutation.^55^, ^103^, ^104^, ^105^, ^106^, ^107^ According to these clinical trials, the median PFS ranges of gefitinib and erlotinib were 9.2–10.8 and 9.7–13.7 months, respectively, whereas the median PFS of platinum‐based chemotherapy ranges from 4.6 to 6.3 months. Therefore, gefitinib and erlotinib are recommended by the U.S. FDA as the first‐line therapy for *EGFR* sensitizing mutation‐positive advanced NSCLC patients in 2009 and 2013, respectively. There is no significant difference between the two EGFR TKIs in the therapeutic effects based on the results of randomized phase III trials.^108^, ^109^ Icotinib, another first‐generation EGFR TKI, has been approved by NMPA in 2011 for the second‐line treatment of advanced NSCLC patients with *EGFR* mutation. The results of ICOGEN trial in China, a double‐blind, head‐to‐head phase III study containing 399 patients, showed that the median PFS of icotinib arm was 137 days as compared to that of gefitinib arm was 102 days.^56^ In 2014, icotinib was recommended as first‐line treatment for advanced NSCLC with sensitizing EGFR mutations. Later, in 2020, the indication of icotinib was expanded by NMPA, as an adjuvant therapy for stage II–IIIA patients who harbor sensitizing *EGFR* mutation. This approval was based on the results from EVIDENCE trial, in which icotinib showed an improved median DFS of 46.9 months compared with 22.1 months in standard chemotherapy group.^110^ Icotinib is now under evaluation by the U.S. FDA for the treatment of *EGFR* mutation‐positive NSCLC patients.

Afatinib and dacomitinib, two irreversible ERBB‐family (pan‐HER) inhibitors, are the second‐generation EGFR TKIs. Unlike the first‐generation, they bind to ATP‐binding domain of EGFR irreversibly and less selectively. Based on the results of three clinical trials, LUX‐Lung 2, LUX‐Lung 3, and LUX‐Lung 6, afatinib showed better ORR (approximately 70%) and prolonged the PFS (approximately 1 year) in advanced NSCLC patients with *EGFR* mutations compared with platinum‐based chemotherapy.^57^, ^58^, ^59^ In LUX‐Lung 6, 364 patients with EGFR mutations were enrolled. The ORR in afatinib arm was 67% compared to 23% in cisplatin‐based chemotherapy arm, and the PFS was prolonged by afatinib (11 vs. 5.6 months).^59^ Afatinib was initially approved by the U.S. FDA in 2013 for the treatment of advanced NSCLC patients with *EGFR* exon 19 deletions or exon 21 (L858R) substitution mutations and got a broadened indication as the first‐line treatment of advanced NSCLC patients with nonresistant *EGFR* mutations. Dacomitinib was approved by the U.S. FDA in 2018 as first‐line treatment for advanced NSCLC patients with *EGFR* exon 19 deletion or exon 21 L858R substitution mutations. The approval was based on a randomized, multicenter, and open‐label clinical trial (ARCHER 1050), in which the median PFS was 14.7 and 9.2 months in the dacomitinib and gefitinib arms, respectively.^60^ However, because of the irreversible and wide binding to ERBB family, the second‐generation EGFR TKIs harbor both better therapeutic effects and more toxic effects than first‐generation TKIs. The advantages of the second‐generation EGFR TKIs over first‐generation EGFR TKIs are not unclear so far.^111^, ^112^

Nearly, all patients received first‐ or second‐generation EGFR TKIs treatment eventually acquired drug resistance, leading to disease progression.^113^ Osimertinib has emerged as a third‐generation EGFR TKI and selectively and irreversible targets to original *EGFR* sensitizing mutations and T790M mutation. T790M is the most common second mutation causing drug resistance, which is referred to a threonine‐tomethionine substitution on codon 790 in exon 20 (T790M).^114^, ^115^ The third‐generation EGFR TKIs, including rociletinib,^116^ PF‐06747775,^117^ olmutinib (HM61713),^118^ nazartinib (EGF816),^119^ avitinib,^120^ osimertinib (AZD9291), aulmonertinib (HS‐10296), and furmonertinib (alflutinib, AST2818), were designed to overcome the T790M mutation. Osimertinib was initially approved for the treatment of advanced NSCLC *EGFR* T790M mutation‐positive patients with disease progression on or after EGFR TKI therapy. Approval went through an accelerated process based on the promising results of AURA extension and AURA 2.^121^, ^122^ The T790M detection rates in the two trials were 64% and 63%, respectively, and the ORRs were 57% and 61%, respectively.^123^, ^124^ Subsequently, based on the results of two clinical trials, FLAURA (NCT02296125) and ADAURA (NCT02511106), the U.S. FDA broadened the indications of osimertinib as the first‐line treatment for advanced NSCLC patients with sensitizing *EGFR* mutations or as an adjuvant therapy when surgery patients with sensitizing *EGFR* mutations.^61^, ^62^ Meanwhile, osimertinib showed advantages on the ability to cross blood–brain barrier, making it possible to enter central nervous system (CNS) and kill tumor cells.^125^ Aulmonertinib (HS‐10296) is the second third‐generation EGFR TKI approved in China. In March 2020, it was approved by the Chinese National Medical Products Administration (NMPA) for the treatment of advanced NSCLC patients with *EGFR* T790M, who got disease progression on or after EGFR TKI therapy. This approval was based on the findings of an open‐label phase II study, APOLLO, in which the median PFS and ORR of patients with progressed NSCLC harboring *EGFR* T790M mutation treated with aulmonertinib was 12.3 months and 68.9%. Of note, aulmonertinib induced an ORR of 61.5% in patients with CNS metastasis.^63^ In 2021, the head‐to‐head phase III clinical trial (AENEAS) compared aulmonertinib with gefitinib as the first‐line treatment in advanced patients with sensitizing *EGFR* mutations. Aumolertinib significantly improved PFS to 19.3 months compared to 9.9 months in gefitinb arm. As for side effects, the incidence of rash or diarrhea was markedly decreased in aumolertinib arm.^126^ Recently, furmonertinib, the third third‐generation of EGFR TKI, was approved by NMPA and shared the same indications with aulmonertinib. Findings of a phase IIb clinical trial were released in ASCO 2020, *EGFR* T790M mutation‐positive advanced NSCLC patients treated with furmonertinib showed an ORR of 74.1% and PFS of 9.6 months.^127^ The ORR and PFS of patients with CNS metastasis were 66% and 11.6 months.^64^, ^128^ Aulmonertinib and furmonertinib are both under clinical investigation on first‐line treatment for advanced NSCLC patients harboring sensitizing *EGFR* mutations.

Targeted therapy strategy contains two main approaches: mAbs and small‐molecule inhibitors (SMIs). The advantages and disadvantages between mAbs and SMI have been well discussed in Ref. 90.^129^ Necitumumab is a second‐generation, recombinant human IgG1 mAb that binds to EGFR, preventing receptor activation. In the phase III clinical trials (SQUIRE), necitumumab in combination with gemcitabine and cisplatin prolonged the OS and PFS of patients with advanced squamous cell lung cancer for 1.6 and 0.2 months, respectively.^66^ Though the benefits were not extraordinary, based on the limited therapy for squamous cell lung cancer patients, the U.S. FDA approved necitumumab combination therapy as the first‐line treatment for advanced squamous NSCLC. However, only patients with EGFR expression (EGFR > 0) would benefit from this limited therapeutic effect.^130^ Other mAbs targeting EGFR, such as cetuximab, nimotuzumab, and panitumumab, have not been approved for the treatment of NSCLC.

At present, EGFR TKIs have been approved for the treatment of sensitizing *EGFR* mutations, exon 19 deletions (Del19) and the L858R point mutation, which account for about 85% of observed *EGFR* mutations in NSCLC.^102^ However, there have been about 600 types of EGFR mutations reported, of which 93% are represent in the exons 18–21, the first four exons for the expression of tyrosine kinase domain.^131^ Limited by the testing methods, only some of them could be detected with high sensitivities, including G719A/S/C, Del19, S768I, exon 20 insertions (Ins20: V769_D770insASV, D770_N771insG/SVD, and H773_V774insH), T790M, L858R, and L861Q.^131^ For now, relevant data on the clinical features of rare *EGFR* mutations have been accessed via post‐hoc analyses of clinical trials and discussed in Ref. 94.^132^ The first‐generation EGFR TKIs, gefitinib and erlotinib, are less effective for G719X mutation with an ORR of 36.8% and median PFS of only 6.3 months, compared to Del19 (65.3%) and L858R (67.5%).^133^ One of the second‐generation EGFR TKIs, neratinib, has shown effective inhibition by targeting G719X mutation but limited effects on Del19 or L858R.^134^ A post‐hoc analysis of three clinical trials, LUX‐Lung 2, LUX‐Lung 3, and LUX‐Lung 6, revealed that afatinib was effective for three rare EGFR mutations, G719X, S786I, and L861Q.^135^ Of note, patients with G719X mutation got a prolonged PFS of 13.9 months, which led to a broaden indication of afatinib by the U.S. FDA, for the treatment of advanced NSCLC patients with G719X mutation. Osimertinib as a third‐generation EGFR TKI also showed potential therapeutic effect on G719X mutation.^136^ Other rare mutations, such as exon 19 insertion,^137^ exon 20 insertion,^98^ and EGFR Kinase domain duplication (EGFR‐KDD),^138^ have been investigated by certain preclinical and clinical researches.

During the last 2 years, breakthroughs have been made in targeted therapy for *EGFR* exon 20 insertion, which is associated with poor prognosis.^139^ In 2021, two drugs against *EGFR* exon 20 insertion, amivantamab‐vmjw and mobocertinib, have won the U.S. FDA approval as second‐line treatment for *EGFR* exon 20 insertion‐positive advanced NSCLC patients. Amivantamab‐vmjw, a bispecific mAb targeting EGFR and MET, has shown an ORR of 40% with a median response duration of 11.1 months.^140^ Later, in September, the approval of mobocertinib (TAK‐788) was based on Study 101, in which mobocertinib showed an ORR of 28% with a median response duration of 17.5 months.^68^

### 3.2 ALK

ALK is an RTK naturally expressed in human tissues, such as brain, small intestine, and testis, without complete understanding of its function, but shows strong oncogenic effects.^141^ Several ligands for ALK have been identified in recent researches, such as FAM150 and heparin.^142^, ^143^ ALK is highly expressed in the nervous system of neonatal brain, but barely expressed in adults.^144^ The expression of ALK is time and spatially controlled. ALK fusion proteins are usually found as oncogenic driver in various malignancies. The first identification of *ALK* as a fusion gene partner was found in anaplastic large‐cell lymphoma in 1994.^145^ *ALK* rearrangements in NSCLC have been found in 2007 by the initial discovery of a fusion gene containing parts of the echinoderm microtubule‐associated protein‐like 4 (*EML4*) gene and *ALK* gene.^146^ There are more than 20 variants of *ELM4‐ALK* fusion identified and *EML4‐ALK*, variant 1 is the most common and well‐studied one.^147^ The *EML4* and *ALK* genes are both located on chromosome 2p, and the variants of *EML4‐ALK* fusion depend on the fusion breakpoint in the *EML4* gene, with the breakpoint in *ALK* is usually at exon 20.^147^ Of note, fusion partners of *ALK* are more than *ELM4*, others like *huntingtin‐interacting protein 1 (HIP1)*,^148^ *kinesin family member 5B (KIF5B)*,^149^ *kinesin light chain 1 (KLC1)*,^150^ *translocated promoter region (TPR)*,^151^ and so on have also been identified in human lung cancer. *ALK* arrangement‐driven tumors account for about 5% of NSCLC, and most of them are Asian, men, and never/light smokers and are likely to be adenocarcinomas.^152^, ^153^ Unlike the predictive effects of some *EGFR* mutations, such as exon 19 deletion is associated with better prognosis, it is not clear whether specific genetic alteration in *ALK* mutation is associated with therapeutic response.

Five ALK TKIs, including crizotinib, ceritinib, alectinib, brigatinib, and loralatinib, have been approved as targeted therapy for advanced NSCLC patients who are confirmed to have *ALK* arrangements.^152^ Crizotinib, a first‐generation ALK TKI, is an oral multiple‐target agent, targeting ALK, ROS1, and MET, and initially developed to target cMET.^154^ Based on the ORRs of 50% and 61% and the PFS of 41.9 and 48.1 weeks in two single‐arm phase I and II clinical trials (expansion cohort of PROFILE 1001 and PROFILE 1005), crizotinib has been approved by the U.S. FDA in 2011 for advanced NSCLC patients with *ALK* rearrangements.^155^

Ceritinib (LDK378) and alectinib are two second‐generation ALK TKIs approved by the U.S. FDA for NSCLC patients failed in or tolerate on crizotinib therapy.^147^ They have shown an obvious advantage of penetrance into CNS compared with crizotinib.^156^ Ceritinib could effectively inhibit certain ALK alteration patterns, such as L1196M, G1269A, I1171T, and S1206Y mutations, but could not overcome two crizotinib‐resistant *ALK* mutations, G1202R and F1174C.^157^ In a randomized, open‐label, phase III clinical trial (ASCEND‐4), ceritinib, as first‐line therapy, showed an ORR of 72.5% and a prolonged median PFS of 16.6 months compared with platinum‐based chemotherapy and the median CNS response duration was 16.6 months in ceritinib arm.^71^ Therefore, the U.S. FDA broadened ceritinib indication to previously untreated *ALK*‐positive advanced NSCLC in May 2017. Alectinib, another second‐generation ALK TKI, has shown an ORR of approximately 50% of patients failed in crizotinib therapy, with median PFS of more than 8 months. A phase III clinical trial (ALEX) revealed that patients treated with alectinib showed prolonged PFS of 26 months compared with 10 months in crizotinib group. The ORR was 79% for alectinib and 72% for crizotinib.^158^

Brigatinib is a small molecular inhibitor targeting both EGFR and ALK, which has been confirmed to overcome the osimertinib‐resistant C797S mutation and expected to be the next‐generation EGFR TKI.^159^ Brigatinib was initially approved for the second‐line treatment for advanced *ALK*‐positive NSCLC patients.^160^ The first‐line efficacy of brigatinib was confirmed by a randomized phase III trial, ALTA1L. Two hundred and seventy‐five previously untreated advanced *ALK*‐positive NSCLC patients were enrolled in this trial. The median PFS and ORR for patients treated with brigatinib was 24 months and 74%, respectively, compared with 11.1 months and 62% for those treated with crizotinib, respectively.^161^ With longer follow‐up, patients with brain metastasis have benefited more from brigatinib treatment compared with crizotinib treatment.^161^ In preclinical studies, brigatinib showed potential to overcome the ceritinib‐ or alectinib‐resistance mutations, including G1202R, F1174C/V, and I1171N/T/S.^162^

Recently, in March 2021, a former second‐line ALK TKI, lorlatinib, has been approved as a first‐line treatment for advanced *ALK*‐positive NSCLC patients.^76^ Lorlatinib is a third‐generation ALK TKI and can target multiple RTKs, including ALK and ROS1, and gets ability to overcome *ALK* and *ROS1‐*resistance mutation, such as I1171T and G1202R.^163^, ^164^ The emergency of lorlatinib brings a breakthrough to the targeted therapy for NSCLC patients with *ALK* mutations. Lorlatinib has shown obvious advantages, including penetration into CNS, fewer side effects, and less drug resistance, compared with former‐generations ALK TKIs.

### 3.3 ROS1

ROS1 is an oncogenic RTK of insulin receptor family encoded by the *ROS1* gene on chromosome 6q22. The biologic function of wild‐type ROS1 is not well identified and there has no specific ligand of ROS1 been found.^165^ The oncogenic effects of ROS1 are based on the constitutively phosphorylated and activated by the fusions with partner genes, such as *CD74* (most common),^166^ *FIG* (fused in glioblastoma, the oncogenic effect of *ROS1* rearrangements first identified),^167^ *SLC34A2* (so lute carrier family 34 member 2),^168^ and so on. For now, 16 genes have been identified as *ROS1* fusion partner genes according to Catalogue of Somatic Mutations in Cancer (COSMIC) data bese, and *ROS1* arrangements have been observed in 1–2% NSCLC, most of which are adenocarcinomas, female, and never or light smokers.^24^ The gold standard for *ROS1* fusion detection is FISH assay.^69^

The *ROS1* amide acid sequence shares 49% homology with ALK in the kinase domain and 77% homology at the ATP‐binding area.^169^ Almost all ALK TKIs showed activity to ROS1. Crizotinib, approved for the treatment of *ALK*‐positive NSCLC, is one of the two targeted agents approved by the U.S. FDA in 2016 for *ROS1*‐positive advanced NSCLC. This approval was based on a single‐arm study in 50 advanced NSCLC patients with *ROS1* arrangements. The ORR was approximately 66% and the PFS was 18.3 months.^69^ Entrectinib (Rozlytrek), the other one approved ROS1 TKI, has been simultaneously approved for the treatment of advanced *ROS1*‐positive NSCLC and *neurotrophic tyrosine receptor kinase (NTRK)* gene fusion‐positive solid tumor, including NSCLC in August 2019. The ORR of advanced NSCLC patients treated by entrectinib was 78% and the response duration was 24.6 months, with ability to penetrate blood–brain barrier.^77^ These results are yielded from three clinical trials, including STARTRK‐2 (phase II), STARTRK‐1 (phase I), and ALKA‐372‐001 trials (phase I).^85^

### 3.4 BRAF

*BRAF* mutations have been observed in 3–8% of NSCLC, most of which are adenocarcinomas and smokers.^170^, ^171^ The most common alteration of *BRAF* mutations (more than half) is a single‐point mutation at residue 600 of exon 15, where valine is replaced by glutamate (Val600Glu, V600E).^170^ The *BRAF* gene encodes a threonine/serine protein kinase, which is involved in MAPK/ERK signaling pathway. V600E mutation leads to uncontrolled activation of BRAF, resulting in abnormal cell proliferation.^172^ Other patterns of *BRAF* mutations can induce either activation or inactivation of BRAF.^173^

One targeted therapy for *BRAF V600E* mutation‐positive advanced NSCLC has been approved by the U.S. FDA in 2017. The therapeutic effects of dabrafenib (BRAF TKI) and trametinib (MEK TKI) combination were confirmed by a nonrandomized, noncomparative, and open‐label trial. The ORRs were 63% and 61% in previously treated patients and treatment‐naive patients, respectively. However, the ORR for patients who received just single agent of dabrafenib was 27%.^174^ The activation of MAPK/ERK signaling pathway is hardly blocked by BRAF inhibitor monotherapy until the addition of MEK inhibitor.^175^ Therefore, the development of BRAF TKI is usually based on the combination of BRAF inhibitors and MEK inhibitors.

### 3.5 KRAS

*KRAS* is an oncogene which belongs to *RAS* GTPase family and in control of crucial cellular pathway, including RAF/MEK/ERKandPI3K/AKT.^176^ *KRAS* mutations are common in NSCLC, accounting for 25% of adenocarcinomas, especially in ever/heavy smokers of western countries.^177^ The genomic aberrations of *KRAS* lead to continuous activation of KRAS and uncontrolled cell proliferation. However, the targeted therapy for KRAS is limited and patients with KRAS mutations have poor prognosis with a median survival of 2.4 years.^178^ Mutations happened in codon 12 or 13 have been studied extensively, including the most common *KRAS* alteration, *G12C* (a point mutation that guanine is replaced by cysteine).^179^ Specific *KRAS* alterations have been well reviewed in Ref. 143.^179^ Early researchers have found that MEK inhibitors (trametinib and selumetinib) showed benefits for *KRAS*‐positive NSCLC patients in combination with chemotherapy.^180^, ^181^ Recently, an inhibitor directly targeting KRAS, sotorasib, was approved by the U.S. FDA as a second‐line therapy for advanced *KRAS* G12C‐mutated NSCLC in May 2021. The approval for sotorasib (Lumakras) was based on the results of a single‐arm and open‐label phase I/II trial, CodeBreaK 100. *KRAS* G12C‐positive advanced NSCLC patients whose disease had progressed on or after chemotherapy were enrolled. The ORR was 36% with a median duration of 10 months.^182^

### 3.6 RET

*RET* encodes a tyrosine kinase receptor on cell surface, which is involved in several crucial signaling pathways, including MAPK, PI3K, JAK/STAT, PKA, and PKC pathways.^183^ *RET* arrangements (*RET* fusion) have been observed in 1–2% NSCLC, mostly adenocarcinomas, never/light smokers, and younger patients.^184^ Numerous fusion partners of *RET* have been identified, among which the intron 15 of *KIF5B* is the most common and widely studied for targeted therapy.^185^ The development of RET targeted therapy is based on multitargeted TKIs. A retrospective analysis of multicenter clinical trial containing 165 *RET*‐positive NSCLC patients showed that the response rates (partial or complete) to cabozantinib, vandetanib, and sunitinib were 37%, 18%, and 22%, respectively. Further responses were observed with lenvantinib and nintedanib‐treated patients.^186^ Multitargeted TKIs showed limited therapeutic effects on *RET*‐positive NSCLC, and several RET‐specific agents have entered clinical trials. Selpercatinib (LOXO292), pralsetinib (BLU‐667), BOS172738,^187^ and TPX‐0046^188^ are highly selective RET targeted agents undergoing clinical trials, with the ability to penetrate into CNS and overcome the acquired resistance on multitargeted TKIs.^189^, ^190^ Recently, based on two phase I/II trials (NCT03157128 and NCT03037385), selpercatinib (Retevmo) and pralsetinib (Gavretotm) have been approved for *RET*‐positive advanced NSCLC.^80^, ^81^ In patients treated with selpercatinib, those previously received chemotherapy had an ORR of 64%, whereas those previously untreated had an ORR of 91%. The median duration was at least 6 months.^80^ The efficacy of pralsetinib was evaluated in 114 advanced NSCLC patients. The ORRs of 87 previously treated and 27 previously untreated patients were 61% and 70%, respectively.^81^

### 3.7 MET

The *MET* (mesenchymal‐epithelial transition factor) gene encodes hepatocyte growth factor receptor, the phosphorylation of which leads to the activation of several cellular signaling pathways, including MAPK, PI3K, STAT, and so on.^191^ *MET* amplification is usually associated with EGFR TKIs resistance. *MET* exon 14 skipping is the most common pattern of *MET* mutations, which leads to increased stability and continuous activation of MET protein.^192^ This mutation has been observed in 3–4% LUAD, and the median age is older than *EGFR*‐ or *KRAS*‐positive NSCLC patients.^193^ After the treatment of chemotherapy, the prognosis of *MET* exon 14 skipping‐positive patients is much poor than driver gene‐negative patients with a median OS of 6.7 versus 11.2 months, which makes MET targeted therapy important.^194^ The MET targeted therapy has made a progress on targeting *MET* exon 14 skipping. Crizotinib, as an MET, ALK, and ROS1 multitargeted inhibitor, was approved for the second‐line treatment of *MET* exon 14 skipping‐positive NSCLC patients.^195^ Capmatinib (Tabrecta, 2020) and tepotinib (Tepmetko, 2021) are two MET TKIs approved by the U.S. FDA for the treatment of *MET* exon 14 skipping mutation‐positive advanced NSCLC. The efficacy of capmatinib was demonstrated in a phase II trial enrolling patients with confirmed *MET* exon 14 skipping. Previously untreated patients benefited more from capmatinib treatment with an ORR of 68% and a median duration of 12.6 months, compared with 41% and 9.7 months in previously treated patients.^82^ The approval of tepotinib was based on the ORR and duration data from a phase II study, VISION. The ORR of both treatment‐naïve and previously treated patients is about 56%, with median duration of 10.8 and 11.1 months, respectively.^83^ In 2021, NMPA approved the first MET TKI in China, savolitinib, which was recommended as the first‐line treatment for advanced NSCLC with *MET* exon 14 skipping mutation. The ORR of savolitinib‐treated group was 42.9%, with a median PFS of 6.8 months and a median OS of 12.5 months.^84^

### 3.8 NTRK

*NTRK (neurotrophic tropomyosin‐related kinases)* genes 1, 2, and 3 encode tropomyosin receptor kinases (TRKs) A, B, and C, respectively. *NTRK* fusion has been observed in 1–2% of NSCLC patients and assumed as targetable mutations.^196^ Larotrectinib, entrectinib, and repotrectinib were approved by the U.S. FDA for the treatment of *NTRK* gene fusion‐positive solid tumor (including NSCLC), who have progressed on their primary treatment or have no satisfactory standard therapy.^88^, ^126^, ^197^ Approval for larotrectinib was based on the data from three multicenter, open‐label, single‐arm clinical trials, LOXO‐TRK‐14001 (NCT02122913), SCOUT (NCT02637687), and NAVIGATE (NCT02576431).^198^ Whereas the approval for entrectinib was based on other three studies, ALKA, STARTRK‐1 (NCT02097810), and STARTRK‐2 (NCT02568267).^85^

### 3.9 HER2

HER2 (ERBB2), with EGFR, HER3, and HER4, belongs to ERBB RTK family. *HER2* amplification and overexpression have been observed in approximately 30% and 10% of lung cancer, respectively.^199^, ^200^, ^201^ However, the development of HER2 targeted therapy is mainly against *HER2* driving mutations, which is rare and present in about 2–3% of patients, mostly women, never/light smoker, and adenocarcinoma.^199^, ^202^ Like *EGFR* mutation, the common mutations in *HER2* occur in exon 20 by insertion of DNA bases.^203^ Many targeted agents, including antibodies (transtuzumab and pertuzumab), antibody–drug conjugates (ADCs), and small molecular TKIs (lapatinib, afatinib, dacomitinib, neratinib, poziotinib, and pyrotinib), have been investigated in clinical trials as monotherapy or in combination of chemotherapy. ADCs, including ado‐trastuzumab emtansine (Kadcyla) and trastuzumab‐deruxtecan (Enhertu), showed most encouraging therapeutic effects for HRE2 mutation‐positive NSCLC patients.^204^, ^205^ Therefore, in 2020, fam‐trastuzumab deruxtecan‐nxki (Enhertu), as second‐line treatment, was granted a breakthrough therapy designation (BTD) for the treatment of patients with advanced NSCLC.^204^, ^206^

### 3.10 VEGF/VEGFR

Besides targeting genomic alterations, inhibition tumor vascular formation by targeting angiogenic factors is also a promising and classic anticancer strategy. Vascular supply is essential for the growth and progression of solid tumor, without which tumors remain stable and localized.^207^ High density of microvessels is associated with poor prognosis and metastasis of NSCLC.^208^ Vascular endothelial growth factor (VEGF) and the interaction with its receptors is thought to be the most potent factor in regulating angiogenesis and is able to enhance the vascular permeability.^209^ Inhibition of tumor angiogenesis is assumed as a promising therapeutic strategy. The antiangiogenic treatment for NSCLC contains mAbs, small‐molecule TKIs, and recombinant human endostatin. Of note, the efficacy of single‐agent antiangiogenic agent is limited, therefore, the recommendation of antiangiogenic treatment for NSCLC is usually based on combination therapy (discussed in combination therapy part).

Bevacizumab (Avastin, Genentech) and ramucirumab (Cyramza, Eli Lilly and Company) are two mAbs approved by the U.S. FDA for the treatment of NSCLC. Bevacizumab, which is the first antiangiogenic drug, inhibits angiogenesis through binding and neutralizing all VEGF isoforms.^210^ The efficacy and safety of bevacizumab in combination with chemotherapy for advanced NSCLC was evaluated in a phase II trial, in which the addition of bevacizumab showed an increased response rate (31.5% vs. 18.8%) compared with chemotherapy alone.^211^ Later, in 2006, bevacizumab in combination with carboplatin and paclitaxel was approved by the U.S. FDA for first‐line treatment of advanced nonsquamous NSCLC. This approval was based on the data from a phase III study (E4599) in which bevacizumab increased the median survival from 10.3 to 12.3 months, making an impressive improvement for NSCLC at that time.^212^ In 2018, bevacizumab in combination with immunotherapy and chemotherapy was approved for first‐line treatment of nonsquamous NSCLC (discussed in combination part). Another monoclinal antibody, ramucirumab (IMC‐1121B), is a fully human IgG designed to bind extracellular VEGF‐binding domain of VEGFR‐2, resulting in the inhibition of angiogenesis.^213^ Ramucirumab received first approval of the U.S. FDA in 2018, in combination with docetaxel for previously treated metastatic NSCLC by improving median survival from 9.1 to 10.5 months.^214^ The U.S. FDA expanded the indication for ramucirumab in 2020 based on the results of RELAY study. Ramucirumab in combination with erlotinib significantly improved the survival of advanced NSCLC with sensitizing *EGFR* mutations (19.4 vs. 12.4 months) compared with erlotinib single‐agent group.^215^

TKIs targeting VEGF/VEGFR, platelet‐derived growth factor/receptor (PDGF/PDGFR), fibroblast growth factor/receptor (FGF/FGFR), and c‐Kit demonstrate effects on inhibition angiogenesis, including sorafenib, sunitinib, vandetanib, nintedanib, and anlotinib.^216^, ^217^, ^218^, ^219^ However, only a few TKIs, including nintedanib and anlotinib, have shown positive anticancer effects. The indication of nintedanib for NSCLC has been approved in Europe but failed in America.^220^ Anlotinib (Focus V, Chia‐Tai Tianqing Pharmaceutical and Advenchen Laboratories) harbors a broad spectrum of targets, including VEGFR 2/3, FGFR1‐4, PDGFR α/β, c‐Kit, and Ret, which is assumed to have strong effects on antiangiogenesis.^221^ Anlotinib was first approved by NMPA as third‐line treatment for advanced NSCLC, which is based on the data from ALTER0302 trial.^222^, ^223^ This approval offered a novel approach for advanced NSCLC patients whose disease progressed after two lines treatment. In 2019, the indication of anlotinib was expanded to third‐line treatment for small cell lung cancer (SCLC), which was based on the ALTER 1202 study.^224^

The angiogenesis process is also negatively regulated by endostatin, which is thought to be a potential target in antiangiogenic strategy.^225^ Endostar (YH‐16), a modified recombinant human endostatin, is the only endostatin applied in clinical use for the treatment of advanced NSCLC. The approval by NMPA was based on a phase III clinical trial in which Endostar in combination with vinorelbine and cisplatin showed increased ORR of 35.4% compared with 19.5% of chemotherapy group.^226^ In a phase III trial, Endostar in combination with cisplatin showed increased ORR (63% vs. 46.39%) in NSCLC with malignant hydrothorax and ascites compared with cisplatin alone.^227^ However, the efficacy of Endostar still requires further validation.^228^

## 4 MECHANISM AND REVERSE OF TARGETED THERAPY RESISTANCE

Since the first TKI, gefitinib, has been introduced to treat NSCLC in the late 1990s, the development of the therapeutic strategies for NSCLC progressed rapidly. Despite the promising effects of TKIs, unavoidable drug resistance has been observed in most patients. The increased heterogeneity within the tumor during targeted therapy is associated with poor therapeutic effects.^229^ The potential mechanism includes secondary mutations, alternative activation through another pathway, and histological and phenotypic transformation.^230^, ^231^ Recent studies showed that late‐generation EGFR or ALK TKIs as the first‐line therapy for NSCLC with EGFR or ALK mutations presented an improve outcome.^60^, ^158^, ^232^ Meanwhile, a repeated molecular profiling at progression is necessary for further treatment decision.

### 4.1 EGFR

Almost all patients have acquired resistance after first‐ or second‐generation EGFR TKIs treatment. The median duration time is less than 1 year.^113^ The most common resistance (more than half) for first‐ and second‐generation EGFR TKIs is due to a secondary mutation of the gatekeeper, Thr790Met (T790M).^115^ T790M mutation is referred to the point mutation at a conserved gatekeeper threonine residue within the ATP‐binding pocket, which is replaced by methionine.^233^ EGFR with T790M mutation shows elevated activation either alone or in combination with primary *EGFR*‐sensitizing mutations in exon 19 or 21.^234^, ^235^ Though T790M mutation causes resistance to early‐generation EGFR TKIs, it is associated with slower tumor growth and better prognosis.^236^ The third‐generation EGFR TKI, osimertinib, is responsible to overcome the T790M as well as sensitizing mutations of *EGFR*. However, resistance to osimertinib has also been observed in clinic. The most common secondary mutation related to osimertinib is EGFR‐C797S,^237^, ^238^ which occurs at the covalent binding site of osimertinib and altering osimertinib binding affinity. Other osimertinib‐related mutations, such as EGFR‐G796S/R and EGFR‐L718Q, inhibit osimertinib binding through physical interference.^239^ Some less common mutations, including L792F/H, T854A, D761Y, L747S, and so on, have been also observed in patients treated with osimertinib.^239^, ^240^ Amplification of wild‐type *EGFR* also shows resistance to osimertinib.^241^ The result of the clinical trial, IMPRESS, showed that doublet chemotherapy was not recommended for patients progressed after EGFR TKIs treatment.^242^

Alternative pathway activation is common during the process of drug resistance of EGFR TKIs, including MAPK, PI3K/AKT, JAK‐STAT3, and SRC pathways. For example, acquisition of mutations of *BRAF* (G469A or V600E) in resistance to early or third generation of EGFR TKIs usually results in the reactivation of MAPK pathway.^243^, ^244^ The activation of JAK‐STAT3 pathway occurs at early stage of EGFR TKIs treatment.^245^ However, in an early‐phase clinical trial, the ORR of combined therapy of JAK inhibitor ruxolitinib and erlotinib was less than 5% in patients with resistance to erlotinib.^246^ This combined regimen might be help in early application of EGFR TKIs, but less helpful in the late resistant phase.^247^ The activation of PI3K/AKT pathway and *KRAS* mutation, including mutations in *PIKC3A* and loss of PTEN, is a negative predictor of EGFR TKIs treatment.^248^, ^249^ Therefore, inhibitors for PI3K/AKT pathway in combination of EGFR TKIs have shown combined efficacy.^250^, ^251^ Of note, mutations of PI3K/AKT pathway are not common in ALK or ROS1 TKIs treatment.

### 4.2 ALK

The secondary *ALK* mutations are relatively variable in patients with drug resistance.^252^ It is estimated that after a duration of around 12 months, crizotinib can induce the gatekeeper mutations, including L1196M and C1156Y.^253^ L1196M is present in about 7% of patients resistant to ALK TKIs.^254^ A patient with C1156Y mutation showed resistance to early‐generation ALK TKIs but response to lorlatinib, a third‐generation ALK TKI. However, upon her disease progress, a secondary mutation (L1198F) occurred, which resulted in resistance to lorlatinib, but resensitized the tumor to crizotinib.^255^ Mutations like G1202R, D1203N, S1206, and amplification of *ALK* have shown resistance to crizotinib via physically interfering TKI binding.^252^, ^256^ Increasing the dose of crizotinib has been assumed to overcome the resistance caused by *ALK* amplification.^257^ *ALK*‐G1202R mutation occurs in less than 2% of patients with resistance to ALK TKIs and is assumed to show resistance to almost all approved ALK TKIs.^252^, ^258^ The third‐generation ALK TKI, lorlatinib, has shown activity against *ALK*‐G1202R mutation with an ORR of 44% in a phase III clinical trial.^252^, ^259^ Alectinib, as a next‐generation TKI of ALK, has induced some other *ALK* mutations, including I1171T and V1180.^260^

Activation of MAPK pathway via *KRAS* amplification or *MEK* mutation in patients with resistance to ALK TKIs was observed.^261^, ^262^ Combined therapy of MEK inhibitors and ALK inhibitors for NSCLC patients has been investigated in clinical trials (NCT03087448).

### 4.3 ROS1

ROS1 and ALK share similar structure of tyrosine kinase domains, therefore, many *ROS1* mutations are structural analogues to *ALK* mutations.^263^ However, according to the spectrum data, ROS1‐resistance mutations are less variable than ALK‐resistance mutations, indicating more therapeutic potential of crizotinib as an ROS1 TKI. Patients received crizotinib have been observed to develop gatekeeper mutation of *ROS1* (L2026M).^264^ *ROS1*‐G2032R and *ROS1*‐D2033N are structural analogous of *ALK*‐G1202R and *ALK*‐D1203N mutations, respectively, which show resistance to crizotinib. In a small sample study, *ROS1*‐G2032R is the most common mutation showing resistance to crizotinib.^265^, ^266^ Meanwhile, activation of MAPK pathway is critical in the mechanism of resistance to crizotinib.^267^

### 4.4 Resistance to other TKIs

During the treatment of TKIs of RET, HER2, and MET for NSCLC patients, the medium duration is usually less than 12 months. V804L mutation of *RET* is a gatekeeper mutation and responsible for the resistance to cabozantinib. It is assumed that ponatinib is the most potent RET TKI to overcome the RET TKIs‐associated drug resistance.^268^ An analogous mutation, *HER2*‐C805S, has been reported at resistance to HER2 TKI therapy in HER2‐mutated NSCLC,^269^ which is assumed to affect the binding of HER2 TKIs. Activation of PI3K pathway via mutations in *PIK3CA* has been reported in NSCLC patients with HER2 TKIs resistance, and combined therapy of mTOR inhibitor showed therapeutic response.^270^ The emergence of secondary mutations after treatment of crizotinib has been observed, including D1228N and Y1230C.^271^, ^272^

### 4.5 Off‐target resistance

Secondary mutations other than targeted gene (off‐target resistance) are also common in the process of drug resistance. During the treatment of targeted therapy, alterations of genetic characteristics are usually associated with drug resistance. In NSCLC patients who have progressed on EGFR TKIs, *MET* amplification occurs with 5–20% incidence and leads to resistance to early generation of EGFR TKIs.^230^, ^273^ Combined therapy of EGFR TKIs and MET TKIs in NSCLC patients has been investigated in clinical trials. In NSCLC patients with *MET* amplification and resistance to prior EGFR TKI treatment, MET inhibitor capmatinib combined with gefitinib showed the response rate of about 15%.^274^ An MEK1 mutation has also been reported in a patient with resistance to ALK TKIs, who got response to an MEK inhibitor.^262^ BRAF mutations (BRAF‐G469A or BRAF‐V600E) have been observed in patients with resistance to EGFR TKIs with an occurrence of 1%.^244^ Amplification of HER2, belonged to the same receptor family of EGFR, has also been observed after treatment of EGFR TKIs.^275^ Increased activation of EGFR has occurred in more than 40% of patients progressed on ALK inhibitor crizotinib.^253^ Meanwhile, the occurrence of *EML4‐ALK* rearrangement is associated with resistance to EGFR TKIs.^153^

Besides those targetable mutations in NSCLC, some proteins also show abnormal expression during drug resistance. Increased expression of AXL receptor tyrosine kinase (AXL) has been observed in the samples of NSCLC patients resistant to EGFR, ALK, or RET TKIs treatment, indicating that AXL TKIs might be an alternative for combination therapy in targeted therapy for NSCLC.^276^, ^277^, ^278^ Other abnormally expressed molecules include proto‐oncogene tyrosine‐protein kinase Src (SRC),^279^ insulin‐like growth factor 1 receptor (IGF1R),^280^, ^281^ KIT,^253^, ^282^ and so on.

### 4.6 Histological and phenotypic transformation

In a group of NSCLC patients with resistance to EGFR or ALK TKIs, histological transformation from an NSCLC to a small‐cell lung cancer histology has been observed.^230^ This transformation is assumed to be associated with RB and EGFR loss.^283^ Transformation to a sarcomatoid carcinoma has been reported in cases with resistance to ALK TKIs.^284^ The alterations of some epithelial‐to‐mesenchymal transition‐associated molecules in tumor, including E‐cadherin, vimentin, and so on, lead to the transformation to a more invasive phenotype.^230^

## 5 IMMUNOTHERAPY FOR NSCLC

The emergency of targeted therapy has improved the survival of certain groups of NSCLC patients; however, the 5‐year survival is still not satisfying.^1^ For driver gene‐negative advanced NSCLC patients, platinum‐based chemotherapy only brings a medium PFS of 4–6 months and a medium OS of 10–12 months.^285^, ^286^, ^287^ Since the immunotherapy was introduced in 1990s, breakthroughs have been made in anticancer therapy^288^ (Table 2). In 2015, the U.S. FDA approved the first ICI, nivolumab, for the third‐line treatment of patients with squamous cell lung carcinoma. Present immunotherapy constitutes two major pathways, CTLA‐4 (cytotoxic T lymphocyte antigen 4)/B7 pathway and PD‐1 (programmed death 1)/PD‐L1 (programmed death‐ligand 1, PD‐L1) pathway. Upon the activation of T cells, the expression of immunosuppressive signaling molecules increases, including PD‐1, CTLA‐4, LAG‐3, TIM‐3, TIGIT, VISTA, and CD244.^289^ These T cell coinhibitory pathways restrict the strength and duration of immune response and protect body from immune‐related damage. Tumors exploit these coinhibitory pathways and achieve immune escape.^289^ ICIs increase body antitumor immune effects via blocking the immune checkpoints mentioned above.^290^ Based on the data released from clinical trials, immunotherapy usually showed advantages of OS instead of PFS. For advanced NSCLC patients without EGFR or ALK mutations, immunotherapy brings promising antitumor effects and better prognosis than traditional therapy. Given the essential role of immunotherapy in anticancer treatment, ICIs are arranged to earlier stage of NSCLC as neoadjuvant or adjuvant therapy and have shown promising efficacy.^291^, ^292^ Though patients receive initial benefits from ICIs, most of them develop drug resistance. Combination therapy is thought to be a way to overcome this resistance.^293^

**TABLE 2:** Immunotherapy in driver gene‐negative advanced NSCLC patients

Target	Checkpoint inhibitor	Indications	Status	Company	Clinical trials
PD‐1					
	Nivolumab (Opdivo)	Advanced squamous NSCLC with progression on or after platinum‐based chemotherapy; Advanced NSCLC with progression on or after platinum‐based chemotherapy; In combination with ipilimumab and two cycles of platinum‐doublet chemotherapy for advanced NSCLC	FDA, Mar 2015 (second line) FDA, Oct 2015 (second line) FDA, May 2020 (first line)	Bristol Mayer Squibb	CheckMate 017, NCT01642004^294^ CheckMate 057, NCT01673867^295^ CheckMate 9LA, NCT03215706^296^
	Pembrolizumab (Keytruda)	Advanced NSCLC with PD‐L1 ≥ 50%; In combination with pemetrexed and carboplatin for advanced nonsquamous NSCLC; In combination with carboplatin and paclitaxel (nab‐paclitaxel) for advanced squamous NSCLC	FDA, Oct 2016 (first line) FDA, May 2017 (first line) FDA, Oct 2018 (first line)	Merck Sharp & Dohme	1. KEYNOTE‐042, NCT02220894^297^ KEYNOTE‐010, NCT01905657^298^; 2. KEYNOTE‐021, NCT02039674^299^ KEYNOTE‐189, NCT02578680^300^ 3. KEYNOTE‐407, NCT02775435^301^
	Cemiplimab‐rwlc (Libtayo)	Advanced NSCLS whose PD‐L1 ≥ 50% (Tumor Proportion Score [TPS] ≥ 50%)	FDA, Feb 2021 (first line)	Regeneron	Study 1624, NCT03088540^302^
	Sintilimab (Tyvyt)	In combination with pemetrexed and platinum‐based chemotherapy for advanced nonsquamous NSCLC In combination with gemcitabine and platinum‐based chemotherapy for advanced squamous NSCLC	NMPA, Apr 2020 (first line) NMPA, Jun 2021 (first line)	Innovent Bio and Lilly	ORIENT‐3, NCT03150875^303^ ORIENT‐12, NCT03629925^304^
	Camrelizumab (AiRuiKa)	In combination with pemetrexed and carboplatin for advanced nonsquamous NSCLC	NMPA, Jun 2020 (first line)	Hengrui Pharmaceuticals	CTR20170322; CameL, NCT03134872^305^
	Tislelizumab (BaiZeAn)	In combination with carboplatin and paclitaxel (nab‐paclitaxel) for advanced squamous NSCLC	NMPA, Jan 2020 (first line)	BeiGene	NCT03663205^306^ NCT03594747^307^
PD‐L1					
	Atezolizumab (Tecentriq)	In combination with bevacizumab, paclitaxel, and carboplatin for advanced nonsquamous NSCLC with no EGFR or ALK genomic tumor aberrations Advanced NSCLC whose tumors have high PD‐L1 expression (PD‐L1 stained ≥ 50% of tumor cells [TC ≥ 50%] or PD‐L1 stained tumor‐infiltrating immune cells [IC] covering ≥ 10% of the tumor area [IC ≥ 10%]), with no EGFR or ALK genomic tumor aberrations	FDA, Dec 2018 (first line) FDA, May 2020 (first line)	Genentech	NCT02366143^308^ NCT02409342^309^
	Durvalumab (Imfinzi)	Unresectable stage III NSCLC whose disease has not progressed following concurrent platinum‐based chemotherapy and radiation therapy	FDA, Feb 2018 (adjuvant therapy)	AstraZeneca	PACIFIC, NCT02125461^292^
CTLA‐4					
	Ipilimumab (Yervoy)	In combination with nivolumab and two cycles of platinum‐doublet chemotherapy for advanced NSCLC	FDA, May 2020 (first line)	Bristol‐Myers Squibb	CheckMate 9LA, NCT03215706^296^

Abbreviations: FDA, the U.S. Food and Drug administration; NMPA, Chinese National Medical Products Administration.

### 5.1 Biomarkers for immunotherapy

The expression of PD‐L1 is used to be treated as a biomarker to assess the response of patients to immunotherapy.^310^, ^311^ However, there is no significant correlation between PD‐L1 expression and OS of patients.^294^, ^312^ Combination tumor‐infiltrating lymphocytes with PD‐L1 expression have shown improved predictive effects.^313^, ^314^ Tumor mutational burden (TMB) is another predictive factor, which is associated with T cells activation and improved prognosis.^315^, ^316^, ^317^ High TMB is associated with high tumor antigenicity, which correlates with the efficacy of immune therapy, regardless of PD‐L1 expression.^318^, ^319^, ^320^ The testing for TMB is relatively expensive since it is a novel predictive marker.^321^, ^322^ Despite these predictive factors, combination therapy with immunotherapy still becomes the optimal choice for patients with PD‐L1 expression ≤ 1%.^323^ Nowadays, intestinal commensal microbiota is also used to diagnose and predict prognosis of disease.^324^, ^325^ In melanoma patients received anti‐PD1 therapy, significant differences have been observed in the diversity and composition of gut microbiome between responders and nonresponders.^326^ These biomarkers provide a way to predict the response of patients; however, specific treatment decision should be more precise and personalized.

### 5.2 PD‐1/PD‐L1 pathway

The PD‐1 molecule is mainly expressed on T/B cells, NK, and MDSCs. PD‐L1 and PD‐L2 are two ligands induced by inflammatory signals. Their interactions downregulate the activation of T cells and the production of cytokines.^327^ Excessive induction of PD‐1 and expression of responsive ligands in inflammatory environment will eventually cause the T cell exhaustion.^289^ Many tumors express high level of PD‐L1, including NSCLC, which indicate the blockade of PD‐1/PD‐L1 pathway is a potential therapeutic mechanism.^328^, ^329^ Several mAbs direct to PD‐1 (nivolumab and pembrolizumab) and PD‐L1 (atezolizumab, durvalumab, and avelumab) have been approved for clinical use.

The expression of PD‐L1 is a predictive biomarker for immunotherapy. The original selective criteria of immunotherapy for advanced NSCLC patients were set as patients with PD‐L1 expression in tumor tissue ≥ 50%. In the trial supporting nivolumab for squamous NSCLC, the outcomes were not assumed to be associated with PD‐L1 expression status.^295^ In contrast, the results from KEYNOTE‐001 showed that PD‐L1 expression ≥ 50% was correlated with improved efficacy of pembrolizumab in advanced NSCLC patients.^330^ However, in the later clinical trial, KEYNOTE‐042, the efficacy of pembrolizumab showed no significant difference among patients with various PD‐L1 expressions.^297^ Therefore, the indication of pembrolizumab and nivolumab for advanced NSCLC patients has been expanded to those with PD‐L1 expression ≥1%. The selective criteria for atezolizumab also considered the expression of PD‐L1 in immune cells.^309^ Of note, PD‐L1 expression assays differ according to the specific antibody used.^331^

#### 5.2.1 Anti‐PD‐1 monoclonal antibodies

Nivolumab (Opdivo), a human immunoglobulin G4 (IgG4) mAb targeting human PD‐1, binds PD‐1 with high affinity and blocks the interaction between PD‐1 and PD‐L1/PD‐L2.^332^, ^333^ Nivolumab was initially approved for the treatment of unresectable melanoma in 2014.^334^ In March 2015, nivolumab was approved by the U.S. FDA for the squamous NSCLC patients, who have progressed on or after platinum‐based chemotherapy. This approval was based on the data from a phase III CheckMate 017 trial, in which nivolumab improved overall survival by 3.2 months compared with docetaxel (9.2 vs. 6.0 months).^294^ Later, in another clinical trial, CheckMate 057, nivolumab also showed improved ORR and response duration of nivolumab compared to docetaxel, leading to the expanded approval for nonsquamous NSCLC patients.^295^ In May 2020, based on the data from CHECKMATE‐9LA (NCT03215706), nivolumab plus ipilimumab and two cycles of chemotherapy as first‐line treatment for metastatic NSCLC patients without *EGFR* or *ALK* mutations were approved by the U.S. FDA.^296^

Pembrolizumab (MK‐3475, Keytruda), a highly selective IgG4‐κ isotype mAb against PD‐1, blocks PD‐1/PD‐L1/PD‐L2 pathway via binding to PD‐1.^335^ It initially received accelerated approval from the U.S. FDA for the second‐line treatment of melanoma in 2014, which was similar to nivolumab.^336^ Though nivolumab was approved for advanced NSCLC patients before pembrolizumab, pembrolizumab is the first anti‐PD‐1 drug approved as first‐line treatment for advance NSCLC patients with PD‐L1 TPS ≥50%. The efficacy of pembrolizumab was evaluated in the KEYNOTE‐001 trial (NCT01295827), in which the overall ORR was 19.4% and the median duration of overall survival was 12.5 months.^330^ In 2019, the indication for pembrolizumab was expanded as the first‐line treatment for advanced NSCLC patients with PD‐L1 expression (Tumor Proportion Score [TPS] ≥1%) and no EGFR or ALK mutations. This approval was based on the data from KEYNOTE‐042 (NCT02220894), in which the outcomes of patients showed no significant difference among TPS ≥1%, TPS ≥20%, and TPS ≥50%.^297^

Cemiplimab‐rwlc is the third anti‐PD‐1 antibody approved by the U.S. FDA for the treatment of advanced NSCLC patients with PD‐L1 expression of at least 50%. Based on the results from Study 1624, cemiplimab‐rwlc showed significantly improved PFS (6.2 vs. 5.6 months) and OS (22.1 vs. 14.3 months) compared to those treated with platinum‐based chemotherapy.^302^

There are other three anti‐PD‐1 antibodies approved by NMPA, not by the U.S. FDA, including sintilimab, camrelizumab, and tislelizumab, for the first‐line treatment of NSCLC. The indications of these three ICIs were all referred to combination with chemotherapy for advanced NSCLC with any PD‐L1 expression level. In 2020, based on the data from a phase III trial, ORIENT‐11, sintilimab in combination with pemetrexed and platinum‐based chemotherapy was approved by NMPA for the first‐line treatment of advanced nonsquamous NSCLC.^337^ Later, in 2021, NMPA expanded the indication of sintilimab to first‐line treatment of advanced squamous NSCLC, in combination with gemcitabine and platinum‐based chemotherapy. This approval was based on a phase III trial, ORIENT‐12, in which the 6‐month PFS was 41.4% and ORR was 64.7%.^304^

Camrelizumab is an anti‐PD‐1 antibody approved in China, which has the most indications in antitumor therapy, including classic Hodgkin lymphoma,^338^ NSCLC, esophageal cancer,^339^ and hepatic carcinoma.^340^ In 2020, camrelizumab in combination with pemetrexed and carboplatin was approved by NMPA for the first‐line treatment of nonsquamous NSCLC patients, with a significant improved median OS of 27.9 months.^305^

Tislelizumab was approved for the first‐line treatment of nonsquamous NSCLC in 2020, which was based on the data from a clinical trial, RATIONALE 304.^306^ In 2021, the efficacy of tislelizumab for squamous NSCLC was evaluated in a phase III trial, BGB‐A317‐307, in which tislelizumab in combination with pemetrexed and platinum chemotherapy (either carboplatin or cisplatin) showed improved PFS (7.6 months).^307^ The combined therapy was approved by NMPA for the first‐line treatment of patients with advanced squamous NSCLC in 2021.

#### 5.2.2 Anti‐PD‐L1 monoclonal antibodies

The treatment strategy for targeting PD‐L1 is similar to anti‐PD‐1, but still has the interaction between PD‐1 and PD‐L2, which is assumed to help balance the body inflammatory response.^341^ Based on this theory, the blockade of PD‐L1 is thought to be associated with reduced immune‐related toxicity, such as immune‐related pneumonitis and colitis. However, there are no sufficient data supporting the difference between PD‐1 and PD‐L1 inhibitors.^342^ For now, two anti‐PD‐L1 antibodies, atezolizumab and durvalumab, have entered clinical application for NSCLC patients. Other anti‐PD‐L1 antibodies, such as BMS‐936559, avelumab, and sugemalimab, have been underestimated.

Atezolizumab (MPDL3280A), a human IgG1 mAb, is the first anti‐PD‐L1 antibody approved for NSCLC patients. In 2016, atezolizumab was initially approved as a second‐line treatment for advanced NSCLC patients whose disease has progressed after chemotherapy.^343^, ^344^ Later, in 2018, based on the IMpower150 trial (NCT02366143), atezolizumab in combination with bevacizumab, paclitaxel, and carboplatin was approved for the first‐line treatment of advanced nonsquamous NSCLC without *EGFR* or *ALK* mutation.^345^ In 2020, the U.S. FDA expanded the indication of atezolizumab to first‐line treatment of advanced NSCLC with PD‐L1 expression ≥ 50% as a single agent. The efficacy was evaluated in the IMpower110 trial with median OS of 20.2 months compared to 13.1 months in chemotherapy arm.^309^ Of note, in 2019, atezolizumab in combination with chemotherapy was approved by the U.S. FDA for the first‐line treatment of extensive‐stage SCLC, which was assumed as a breakthrough in SCLC treatment.^346^

Durvalumab (MEDI4736) is a human IgG1 antibody with high affinity of PD‐L1, which was approved by the U.S. FDA as adjuvant treatment of unresectable advanced NSCLC, whose disease has not progressed following concurrent platinum‐based chemotherapy and radiation therapy. This approval was based on the data from a phase III trial, PACIFIC (NCT02125461). Advanced NSCLC completed concurrent chemotherapy and radiation and then received durvalumab or placebo. The PFS in durvalumab group was significantly improved compared with placebo arm.^292^, ^347^

### 5.3 CTLA‐4 pathway

CTLA‐4 is another inhibitory molecule expressed on activated T cells to block the excessive immune response. The responding ligands for CTLA‐4 include CD80 (B7.1) and CD86 (B7.2), which are similar to CD28, an activating signal molecule on T cell surface.^348^ CTLA‐4 inhibits immune response through competitive bind to B7 ligands and blocking the interaction between CD28 and B7 ligands, resulting in decreased activation of T cells.^327^ For now, only one anti‐CTLA‐4 antibody, ipilimumab, has been approved by the U.S. FDA for clinical use. Another anti‐CTLA‐4 antibody, tremelimumab, in combination with other immunotherapy has been investigated in clinical trials.^349^

#### 5.3.1 Anti‐CTLA‐4 monoclonal antibodies

Ipilimumab (BMS‐734106) is a fully humanized IgG1 mAb and was first approved in melanoma treatment with favorable outcomes.^350^ The combination therapy of ipilimumab for NSCLC has been under investigation. A phase II clinical trial was conducted to evaluate the efficacy of ipilimumab in combination with chemotherapy (carboplatin and paclitaxel) in advanced NSCLC. The combination group showed improved median OS (12.2 vs. 8.3 months) and PFS (5.5 vs. 4.6 months) compared with chemotherapy group.^351^ The breakthrough of ipilimumab in the treatment of NSCLC was based on the combination with nivolumab. In 2020, based on the data from a phase III clinical trial, CheckMate 227, nivolumab in combination with ipilimumab was approved by the U.S. FDA for the treatment of advanced NSCLC whose PD‐L1 expression ≥1% and without *EGFR* or *ALK* mutation. The OS of patients in immunotherapy combination arm was 17.1 versus 14.9 months in chemotherapy arm. Of note, the combination of two immunotherapy agents demonstrated more treatment‐related adverse events than single agent, with 76.7% of patients reported.^352^

### 5.4 Other immunotherapy agents

Tiragolumab is an anti‐TIGIT mAb. The data of combination of tiragolumab with atezolizumab in CITYSCAPE study have been released at the ASCO2020 conference. Addition of tiragolumab to atezolizumab demonstrated an increased RR (37.3% vs. 20.6%) and PFS (5.6 vs. 3.9 months) versus atezolizumab alone.^353^ Based on the promising efficacy, in 2021, the U.S. FDA granted tiragolumab BTD in combination with atezolizumab (Tecentriq) for the first‐line treatment of individuals with metastatic NSCLC whose tumors have PD‐L1 expression ≥50% and no *EGFR* or *ALK* genomic tumor aberrations. In late 2021, tiragolumab in combination with dabrafenib, a BRAF TKI, was approved by the U.S. FDA for the treatment of metastatic NSCLC with *BRAF* V600E mutation. This approval was based on the data from Study BRF113928 (NCT01336634), in which the ORRs in previously treated and untreated group were 63% and 61%, respectively, compared with 27% of dabrafenib single‐agent group.^78^ Other immunotherapeutic agents targeting LAG3, IDO, CD137, and OX40 have been investigated in clinical trials.^354^

## 6 THE COMBINATION THERAPY FOR ADVANCED NSCLC

The rapid development of targeted therapy and immunotherapy has profoundly changed the treatment strategy for NSCLC. Balancing the benefits and risks of various treatments and providing patients with best treatment with less adverse events is important for the treatment decisions. Diversity of combinations have been investigated in clinical trials.

### 6.1 Targeted therapy in combination with chemotherapy

There is limited evidence supporting the efficacy of combination of chemotherapy and targeted therapy. A phase II clinical trial found a PFS benefit (15.8 vs. 10.9 months) of gefitinib plus pemetrexed compared with gefitinib alone, but without statistical significance.^355^ Another study, JMIT, showed both improved PFS and OS in the gefitinib plus pemetrexed and carboplatin group.^356^ NEJ009 study got positive results on PFS, but the OS benefit of chemotherapy and targeted therapy combination required further validation.^357^ Therefore, for those driver gene‐positive NSCLC patients, receiving more treatments during whole disease is more important than receiving combination therapy or simultaneous treatment.

### 6.2 Targeted therapy in combination with immunotherapy

Generally, immunotherapy is less effective than targeted therapy in NSCLC patients with targetable driver gene mutations. Several clinical trials evaluated the efficacy of immunotherapy in previously treated NSCLC patients with driver gene mutations.^295^, ^298^, ^343^ The results showed that there was no significant improvement in OS.^358^ Meanwhile, combination therapy showed increased toxicity, which resulted in the termination of some clinical trials.^359^, ^360^ Thus, targeted therapy in combination with immunotherapy is not the best choice for NSCLC patients with driver gene aberrations, especially for those with *EGFR* or *ALK* mutations, according to the current evidence. In *AXL* gene aberration‐positive NSCLC patients, bemcentinib, an AXL TKI, in combination with pembrolizumab showed significantly improved prognosis and has been granted fast track designation by the U.S. FDA in June 2021 (NCT03184558). Therefore, targeted therapy in combination with immunotherapy might be an alteration for drug development of those immature targets. However, for patients get drug resistance and progress on targeted therapy, immunotherapy plus chemotherapy and antiangiogenesis is a promising strategy to improve survival.^361^ IMpower150 first showed benefits of ICIs on *EGFR* mutation‐positive NSCLC patients, with improved PFS (10.2 vs. 7.1 months) in atezolizumab plus bevacizumab and chemotherapy group compared with bevacizumab and chemotherapy combination group in *EGFR* mutation‐positive NSCLC patients.^345^ Other ongoing studies, like KEYNOTE‐789, Checkmate‐722, ORIENT‐3, and TREASURE, are also investigating the therapeutic effects of immunotherapy plus chemotherapy and antiangiogenesis in *EGFR* mutation‐positive advanced NSCLC.

### 6.3 Immunotherapy in combination with chemotherapy

The combination of immunotherapy and chemotherapy is a new standard therapeutic regimen for advanced NSCLC patients, especially those without driver gene mutation. The basic theory is that increased expression of tumor antigens and PD‐L1 expression in the immunological environment induced by chemotherapy agents might enhance the therapeutic effects of immunotherapy.^362^ Meanwhile, immunotherapy in combination with chemotherapy has demonstrated improved efficacy independent of PD‐L1 expression compared with chemotherapy alone. The efficacy of chemotherapy in combination with immunotherapy is apparent in both advanced squamous and nonsquamous NSCLC, especially those without *EGFR* or *ALK* mutations. Based on the data from clinical trials, different chemoimmunotherapies have been approved by the U.S. FDA for the first‐line treatment of advanced NSCLC.^363^ Several anti‐PD‐1 antibodies approved by NMPA are also in regimens combined with chemotherapy (discussed in anti‐PD‐1 part).

KeyNote 189 is the first clinical trial demonstrating the promising therapeutic effects of chemoimmunotherapy compared with chemotherapy alone in nonsquamous NSCLC, in which pembrolizumab in combination with platinum‐based chemotherapy significantly improved the OS and PFS of NSCLC patients without EGFR or ALK mutations.^300^ Another study, IMpower 130, evaluated the therapeutic effects of platinum‐based chemotherapy in combination with atezolizumab, an anti‐PD‐L1 antibody, in advanced nonsquamous NSCLC. Improve OS (18.6 vs. 13.9 months) and PFS (7 vs. 5.5 months) were observed in the combination group compared with chemotherapy alone.^364^ Chemoimmunotherapy in combination with antiangiogenic agent for the treatment of nonsquamous NSCLC has also been approved by the U.S. FDA based on the data from IMpower 150 study. Carboplatin–paclitaxel–atezolizumab in combination with bevacizumab, an antiangiogenic antibody, has demonstrated an improved OS and PFS.^308^

Studies also have been performed in squamous NSCLC and demonstrated promising outcomes. KeyNote 407 is the first phase III study to make a change in the standard treatment for squamous NSCLC. Carboplatin–paclitaxel or abraxane in combination with pembrolizumab demonstrated a better OS (15.9 vs. 11.3 months) compared with chemotherapy alone.^301^ The efficacy of combination therapy containing anti‐PD‐L1 inhibitor has also been evaluated in another phase III clinical trial, IMpower131. Carboplatin–nab–paclitaxelin in combination with atezolizumab has showed improved PFS (6.5 vs. 5.6 months) compared with chemotherapy alone.^365^

### 6.4 Anti‐PD‐1/PD‐L1 in combination with anti‐CTLA 4

The efficacy of combination of anti‐PD‐1/PD‐L1 pathway with anti‐CTLA4 has been under clinical investigation. As mentioned above, nivolumab in combination with ipilimumab has been approved by the U.S. FDA for the first‐line treatment of advanced NSCLC.^352^, ^366^ Patients in nivolumab and ipilimumab combination group showed increased RR (response rate) of 45% versus 26.9% in chemotherapy group. However, due to high incidence of adverse events, withdrawals of dual immunotherapy are common.^367^ The efficacy of durvalumab in combination with tremelimumab, another anti‐CTLA‐4 antibody, versus chemotherapy has been investigated in ARCTIC study.^368^ Though this combination showed an increase in OS (11.5 vs. 8.7 months), when compared with combination of anti‐PD‐1/PD‐L1 and chemotherapy, the survival data were not satisfying.

### 6.5 Combination with antiangiogenesis strategy

There are two antiangiogenic drugs approved by the U.S. FDA for the treatment of NSCLC. Bevacizumab in combination with chemotherapy (carboplatin and paclitaxel) was initially approved by the U.S. FDA in 2006, for the treatment of advanced nonsquamous NSCLC.^369^ In patients with EGFR mutation, the PFS in erlotinib plus bevacizumab group was 16 months compared with 9.7 months in erlotinib single‐agent group.^370^ In another phase III study, NEJ026, erlotinib in combination with bevacizumab improved PFS from 13.3 to 16.9 months.^371^ However, the benefit of OS requires further validation. In the NEJ026 study, combination therapy (bevacizumab plus erlotinib) also showed an increase in adverse events.^372^ The efficacy of antiangiogenesis in combination with immunotherapy has been confirmed in the IMpower150 study. Atezolizumab plus carboplatin plus paclitaxel plus bevacizumab (ACPB) regimen significantly improved the PFS and OS in advanced NSCLC patients, regardless of PD‐L1 expression and EGFR or ALK genetic alteration status.^308^ Based on the results of IMpower150, ACPB regimen was approved by the U.S. FDA in 2018 for the first‐line treatment of advanced nonsquamous NSCLC with no *EGFR* or *ALK* genomic tumor aberrations. In 2020, the U.S. FDA approved the first anti‐VEGFR and EGFR–TKI combination as first‐line treatment for advanced NSCLC. Ramucirumab (Cyramza), a VEGFR2 antagonist, in combination with erlotinib showed improved survival for patients with *EGFR* exon 19 deletion and L858R mutations. But patients in combination group also demonstrated high incidence (72%) of grade 3–4 treatment‐emergent adverse events.^215^

## 7 OTHER PROMISING THERAPIES FOR NSCLC

### 7.1 HER2 and HER3

HER2 and HER3 belong to an RTK family that includes EGFR (ERBB1), HER2 (ERBB2/NEU), HER3 (ERBB3), and HER4 (ERBB4). In contrast to EGFR, no ligand has been identified for HER2 and only one ligand, heregulin (HRG), for HER3 has been identified. Still, they are involved in receptor interaction and promote the dimerization of all ERBB family components.^373^, ^374^ EGFR TKIs are thought to have potential effects on the mutations of other ERBB family members, which requires further validation.^375^ Afatinib, a second‐generation EGFR TKI targeting ERBB family, did not show therapeutic benefit in NSCLC with *HER2* mutations.^376^ Mobocertinib (TAK788) is dual TKI targeting *EGFR* insertion and *HER2* mutation, which is approved for the treatment of *EGFR* exon 20 insertion mutations. Mobocertinib is granted a BTD from the U.S. FDA for NSCLC with *EGFR* or *HER2* exon 20 insertion mutations, based on the data from a phase II trial.^68^ Despite the approval of fam‐trastuzumab deruxtecan‐nxki (Enhertu), other inhibitor targeting HER2 showed promising effects on NSCLC. Poziotinib, an oral irreversible pan‐HER TKI, demonstrated a promising ORR of 35.1% in *HER2‐*mutated NSCLC in a phase II trial, ZENTITH20‐2.^377^ The safety and effects of another irreversible pan‐HER TKI, pyrotinib, have been investigated in a phase II study and showed tolerable response with an ORR of 31.7%.^378^

The aberrant activation of HER3 is observed in NSCLC and more than 80% of NSCLC patients with *EGFR* mutation express HER3.^379^ Upregulating HER3 in cancer cells is also associated with resistant to EGFR TKIs.^380^ Patritumab deruxtecan (U3‐1402, HER3‐DXd) is an ADC agent consists of an HER3‐targeted antibody and a topoisomerase I inhibitor. HER3‐DXd has shown the most promising effects and tolerable safety in NSCLC with *EGFR* mutations in a phase I clinical trial, with a disease control rate of 70% and median duration of 6.9 months.^381^ The efficacy of HER3‐DXd will be confirmed in a phase II trial, HERTHENA‐Lung01 (NCT04619004). Other mAbs targeting HER3, including patritumab and lumretuzumab, are under evaluation in early clinical trials.^382^, ^383^

### 7.2 PIK3CA

The phosphoinositide 3‐kinase (PI3K) pathway regulates multiple cellular biologic process. The aberrant activation of PI3K/AKT/mTOR signaling is common in different cancer types.^384^ The *phosphatidylinositol‐4,5‐bisphosphate 3‐kinase, catalytic subunit alpha (PIK3CA)* gene encodes the catalytic subunit alpha of PIK3. However, the mutations of *PIK3CA* are not common in lung cancer (mainly observed in LUSC), by contrast amplification of which is more prevalent.^385^ *PIK3CA* mutations are associated with worse OS and PFS in patients with NSCLC.^386^ Targeting PI3K strategies contain selective PI3K inhibitors and PI3K/mTOR inhibitors. Taselisib, buparlisib, voxtalisib, and PX‐866 are PI3K inhibitors under investigation of phase II clinical trial as monotherapy or in combination with other treatments.^387^, ^388^, ^389^ In one study, the addition of PX‐866 showed an increased ORR (6% vs. 0%) compared with docetaxel single agent, whereas with no improvement in PFS or OS (NCT01204099). Apitolisib is a dual PI3K/mTOR inhibitor, the efficacy of which was evaluated in a single‐arm phase Ib trial in combination with carboplatin and paclitaxel and bevacizumab (NCT01301716). Based on the current knowledge, more evidence is required to confirm the therapeutic effects of targeting PI3K.

### 7.3 Epigenetic therapy

Epigenetic alteration is one of the cancer hallmarks, which is mainly referred to DNA methylation, histone deacetylation, and noncoding RNAs (ncRNAs). Epigenetic drugs have made breakthroughs in hematologic malignancies and are approved by the U.S. FDA, including HDAC inhibitors for cutaneous T cell lymphoma (vorinostat and romidepsin), DNMT inhibitors for myelodysplastic syndrome (5‐azacytidine and decitabine), and JAK1/2 inhibitor for myelofibrosis (ruxolitinib). The approval of tazemetostat, an EZH2 inhibitor, for epithelioid sarcomas is a milestone in epigenetic anticancer therapy for solid tumor.^390^

DNA hypermethylation happens during early stage of carcinogenesis and has been widely studied.^391^, ^392^ DNA methyltransferase inhibitor (DNMTi) is capable to reverse the hypermethylation of DNA, especially the hypermethylation of tumor suppressor. Azacytidine and decitabine are two common DNMTi used in clinical trials.^393^ Deacetylation by histone deacetylase (HDAC) leads to tumor suppressor silencing. HDAC inhibitors (HDACi) bind to the catalytic region of HDAC and prevent tumor suppressor silencing. Common HDAC inhibitors include trichostatin A, SAHA, depsipeptide, and valproic acid. ncRNA‐targeted therapy through miRNA is another potential treatment, which is widely investigated in clinical trials.^394^ ncRNAs, including long noncoding RNAs (lncRNAs), short micro‐RNAs, and circular RNAs (circRNAs), are involved in tumorigenesis and tumor progression.^395^ For example, miR‐34a mimic and miR‐16 mimic are currently being tested in phase I clinical trial for multiple solid tumors, including NSCLC.^396^, ^397^ Inhibition of lncRNAs and circRNAs has also been investigated in clinical trials. However, the disadvantages of RNA‐based therapy are apparent, such as off‐target effects, which are urgently required to overcome. Epigenetic therapy can also be used to resensitize cancer cells with resistance to other treatments, for example, resistance to TKIs.^398^

The treatment strategy through regulation of epigenetic events is usually based on combination therapy.^399^, ^400^ Epigenetic therapy in combination with targeted therapy, chemotherapy, and immunotherapy has been investigated in clinical trials. Early trials, such as azacitidine plus erlotinib and decitabine plus cisplatin, showed unsatisfied therapeutic effects.^401^, ^402^ In a phase II study, though entinostat with erlotinib did not show therapeutic advantages, this therapy improved OS in advanced NSCLC patients with high level of E‐cadherin.^403^ This indicated that identification of epigenetic biomarkers is important for application of epigenetic therapy. A recent clinical trial compared pembrolizumab plus azacitidine versus pembrolizumab alone. The combined therapy showed an ORR of 20% versus 14% with median PFS 2.9 versus 4.0 months. Importantly, the incidence of grade≥ 3 toxicity in combination arm was much higher than single‐agent arm (78% vs. 55%).^404^ HDAC inhibitors combined with DNMT inhibitors have also been investigated in clinical trials. A phase II clinical trial combining azacitidine and entinostat showed an OS of 6.4 months in all enrolled advanced NSCLC patients but 10.4 months in “methylation marker”‐positive patients, including *APC, RASSF1A, CDH13*, and *CDKN2A*.^399^ Given the current evidence for epigenetic therapy, more studies are required to confirm the therapeutic effects in NSCLC.

## 8 DISCUSSION AND FUTURE PERSPECTIVE

Surgery provides the best chance to cure the disease, but most NSCLC patients present with advanced‐stage disease. Cytotoxic chemotherapy had held a leading role in the treatment of advanced NSCLC until the first time TKIs became the first‐line treatment in certain subgroups of NSCLC patients. During the last decade, targeted therapy and immunotherapy have changed the treatment strategies for NSCLC, especially those in late phases of this disease. Based on the features of genetic alterations and PD‐L1 expression, clinicians make the final treatment decision more personalized. However, major challenges still remain, including identifying new targetable genetic alterations, developing new drugs and effective drug combinations, overcoming drug resistance, and discovering better biomakers to predict therapeutic response and prognosis of patients. Combination therapy could be an effective strategy in overcoming TKI resistance by targeting the native genomic mutation and the secondary alterations. However, the use of combination therapies increases the drug‐associated toxicity, which requires strict validation. To determine further treatment, molecular testing is not only essential for initiation of treatment, but also necessary at the time that disease progresses.

Currently, a variety of treatment strategies are available for NSCLC. Choosing appropriate and personalized combination therapy for patients is challenging for clinicians. Of note, patients not only benefit from the release of new drugs, but also benefit from new use or combos of the existing drugs.

## CONFLICT OF INTERESTS

The authors declare no competing interests.

## ETHICS STATEMENT

Ethics approval was not required for this review.

## AUTHOR CONTRIBUTIONS

Yuan Cheng: conceptualization, methodology, and writing. Tao Zhang: Figure preparation. Qing Xu: conceptualization and reviewing.

## Data Availability

The datasets in this study are available from the corresponding author on reasonable request.
